# Schematic memories develop quickly, but are not expressed unless necessary

**DOI:** 10.1038/s41598-020-73952-x

**Published:** 2020-10-12

**Authors:** Alexa Tompary, WenXi Zhou, Lila Davachi

**Affiliations:** 1 Department of Psychology, University of Pennsylvania, Philadelphia, PA 19104 USA; 2 Center for Neural Science, New York University, New York, NY 10003 USA; 3 Department of Psychology, Columbia University, New York, NY 10027 USA; 4 Nathan Kline Institute for Psychiatric Research, Orangeburg, NY 10962 USA

**Keywords:** Psychology, Human behaviour

## Abstract

Episodic memory retrieval is increasingly influenced by schematic information as memories mature, but it is unclear whether this is due to the slow formation of schemas over time, or the slow forgetting of the episodes. To address this, we separately probed memory for newly learned schemas as well as their influence on episodic memory decisions. In this experiment, participants encoded images from two categories, with the location of images in each category drawn from a different spatial distribution. They could thus learn schemas of category locations by encoding specific episodes. We found that images that were more consistent with these distributions were more precisely retrieved, and this schematic influence increased over time. However, memory for the schema distribution, measured using generalization to novel images, also became less precise over time. This incongruity suggests that schemas form rapidly, but their influence on episodic retrieval is dictated by the need to bolster fading memory representations.

## Introduction

Decades of work in cognitive psychology has demonstrated that schemas can enhance^1,2^ memory formation. But while the impact of schemas on new memories is well documented, it is less clear both when schema knowledge is solidified (accessibility) and when it begins to exert an influence on episodic memories (expression). In other words, when is a schema formed, and when is it used?.

It is thought that a schema encompasses commonalities across multiple unique experiences, but that the specific details of each experience are lost over time. Indeed, formal definitions of a schema require that it (1) is constructed from multiple episodes, and (2) lacks episode-specific details^3^. How do such structured memories develop? Neuroscientific theories of systems-level consolidation posit that successful retrieval of episodic memories is initially supported by the hippocampus, but, over time, memories are supported by distributed cortical regions through incremental, coordinated reactivation of memories across the hippocampus and cortex^4,5^. This process is thought to underpin the slow extraction and cortical representation of statistical regularities common across overlapping episodes^6^. Richards and colleagues sought evidence for these theories by simultaneously probing episodic memory and schematic memory in a water maze experiment. After 30 days, rodents’ swim patterns more closely matched the learned schema of platform locations, despite weaker memory for specific platforms, consistent with the idea that schematic memory emerges as memory for specific episodes are forgotten^7^. We have shown in prior work that neural patterns in the human hippocampus and medial prefrontal cortex come to reflect overlap across associative memories after a week, which may support the gradual organization of discrete episodes into schematic knowledge^8^. A slowly emerging schematic memory can also explain a wide and diverse family of behavioral observations that both time and sleep benefit generalization across overlapping experiences, including (but not limited to) overlapping episodes, statistical regularities, and category members with shared features^9–11^. Furthermore, after a delay or a night of sleep, schema-consistent episodes are more likely to be remembered^12,13^ and remembered with greater precision^14^ relative to schema-inconsistent episodes.

However, the slow development of schemas is difficult to reconcile with observations that schemas can form rapidly and are used simultaneously with, or soon after, the encoding of its constituent episodes. Indeed, some of the earliest observations of schematic memory come from perception research, operationalizing a schema as a common set of spatial properties across visual displays that participants learn and use within minutes^15,16^. Furthermore, newly developed schemas have been shown to influence novel, schema-consistent memories during and immediately after encoding the memories that make up the schema^12,14,17^. These findings suggest that schemas can develop quickly and in the absence of time-dependent processing, in contrast to predictions of systems-level consolidation models.

**Figure 1:**
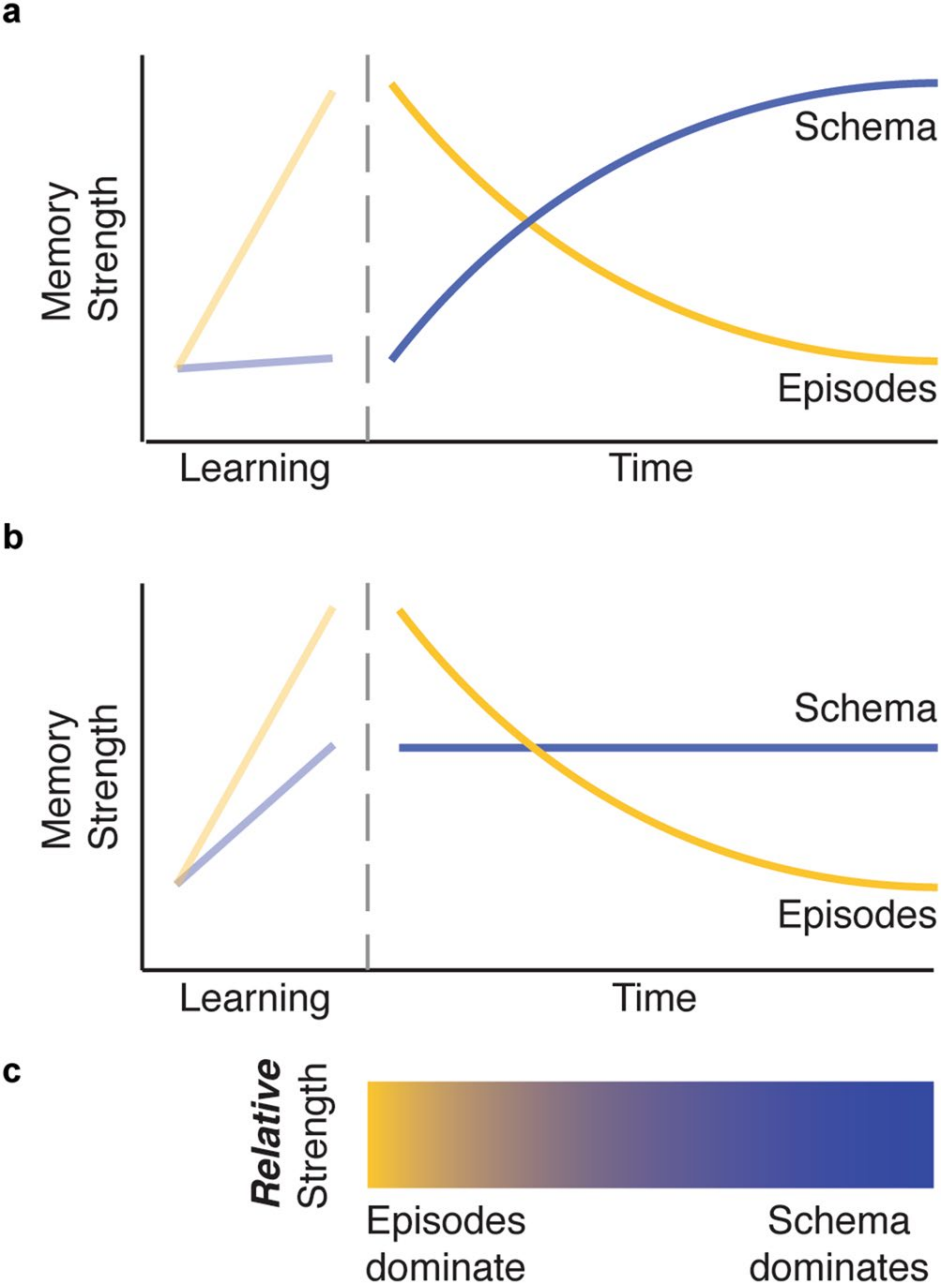
Two hypotheses of time-dependent changes in the retrieval of newly encoded episodic and schematic memories, assuming successful encoding of the episodes. (**a**) After learning its constituent episodes, a schematic memory emerges over time, while the strength of its constituent episodic memories decreases. This emergence is thought to be driven by the slow extraction of overlapping information across different episodes. (**b**) During learning, a schematic memory forms concurrently with the episodes that it comprises. Over time, it remains stable as its constituent memories are forgotten. (**c**) Both hypotheses predict that retrieval of a schema’s constituent episodes would be more affected by the schema over time, indicated by the yellow-blue gradients (bottom).

How might these two different accounts—the slow strengthening of schemas over time, versus their immediate formation and use—be reconciled? One approach is to disentangle the *accessibility* of a schematic memory from its *expression* during episodic retrieval. One hypothesis would predict that episodic memories are accessible immediately, while a schema based on those episodes takes time to develop, thus explaining the increasing influence of schematic representations during episodic retrieval over time (Fig. 1a). A second hypothesis is that both schematic representations and episodic memories develop concurrently during encoding, but episodic memories are forgotten over time while schematic memory remains stable (Fig. 1b). According to this view, both types of memory have formed, but which one is expressed depends on when memory is tested. Soon after encoding, the episodic memories dominate retrieval because they are more strongly remembered. At this point, the schema representation is not as necessary to support precise retrieval, so its influence on retrieval is weaker. However, as the strength of episodic memories decreases, the schematic memory becomes more involved in episodic retrieval.

Both hypotheses predict that schematic memory is more likely to be expressed during episodic memory retrieval after a delay, when episodic memories are relatively weaker (Fig. 1c). However, these hypotheses offer different predictions about the strength of memory for the schemas themselves over time. The first hypothesis predicts weak or no schematic memory early on, while the second hypothesis predicts intact and strong schematic memory. Ideally, a pure assessment of schematic memory would minimize interference from its constituent episodes. One way to do this is by probing how a schema is recruited to make decisions about novel, yet related, information (i.e. generalization). While past work has shown that schemas generalize to new stimuli immediately after encoding, and this generalization is sometimes enhanced after a period of consolidation^9,11,18^, the relationship between generalization of a schema and memory for its constituent episodes is less studied. Quantifying changes in this relationship over time would provide the critical comparison needed to understand the development of a schema separately from its expression during episodic retrieval.

**Figure 2:**
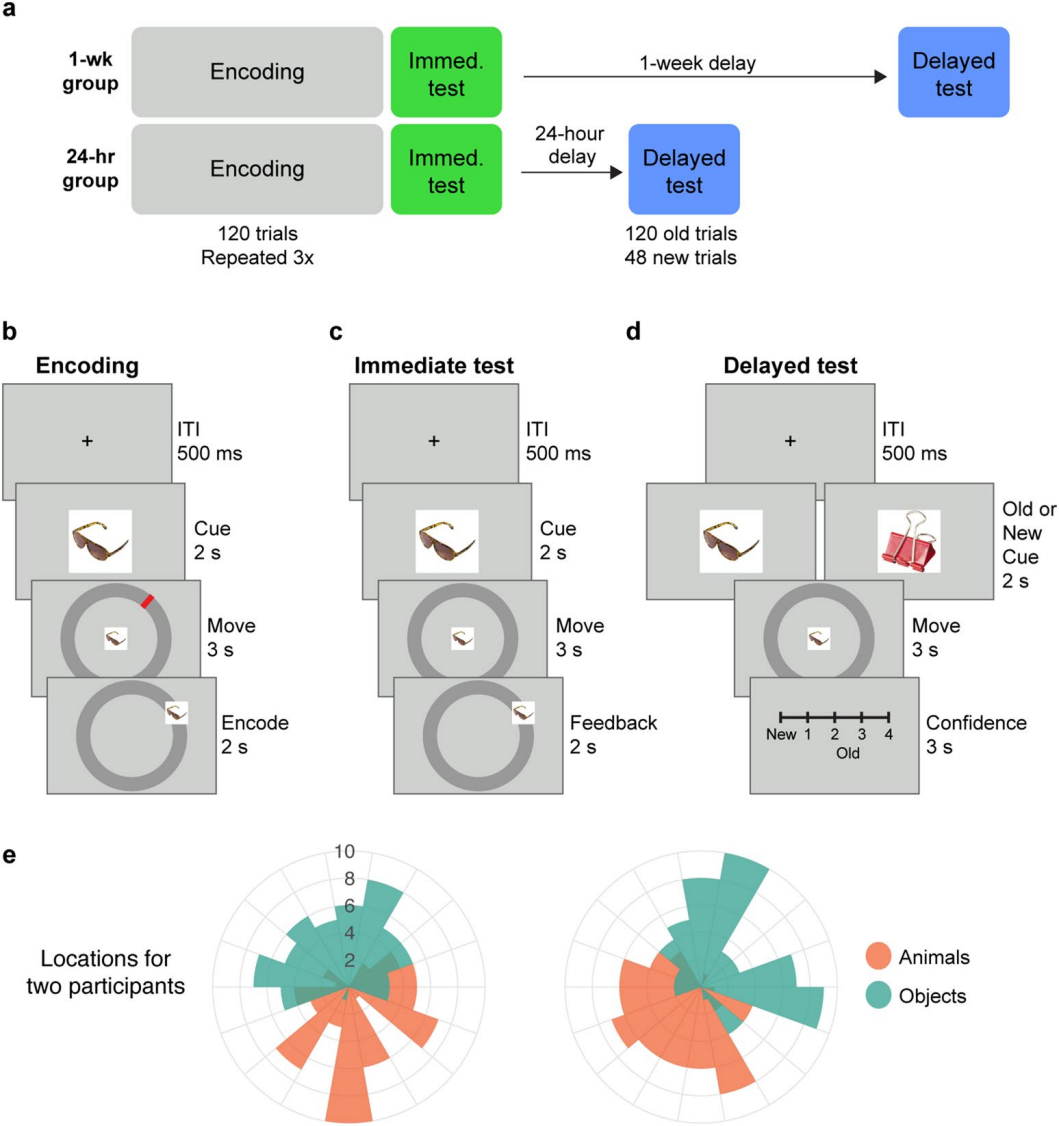
Experiment design. (**a**) In Session 1, participants completed three cycles of image-location encoding, followed by an immediate location memory test. Participants completed a delayed test 1 week after encoding or 24 h after encoding. (**b**) During encoding, participants dragged images onto their associated location on a ring, indicated by a red mark. (**c**) During the immediate test, participants dragged each image onto its retrieved location on the ring. The image then was presented at its correct location. (**d**) The delayed test was identical to the immediate test, but instead of a feedback phase, participants rated their confidence for their location memory. (**e**) Radial plot of distributions of animal and object locations for two participants. The locations of images in each category were determined by sampling from two cosine distributions centered around opposite points on the ring.

In this behavioral experiment, we sought to tease apart the strength of schemas from their influence on constituent episodic memories. Specifically, we developed an experiment to (1) investigate the influence of schematic memory on episodic retrieval over time, as those component memories become less precise, and (2) probe the use of schema knowledge when episodic memory retrieval is not required, using a generalization task with new memoranda. Participants encoded and retrieved associations between images from two categories (animals or objects) and their locations along a ring (Fig. 2a). The locations of images for each category were drawn from a probability function centered at opposite ends of the ring (Fig. 2e). Thus participants could learn both episodic memories (specific image locations) and schematic memories (the likely location of animals and objects). At immediate and delayed retrieval tests, participants were probed for the location of each image (Fig. 2c, d). Delayed retrieval was tested either 24 h or 1 week after encoding in separate groups of participants.

**Figure 3:**
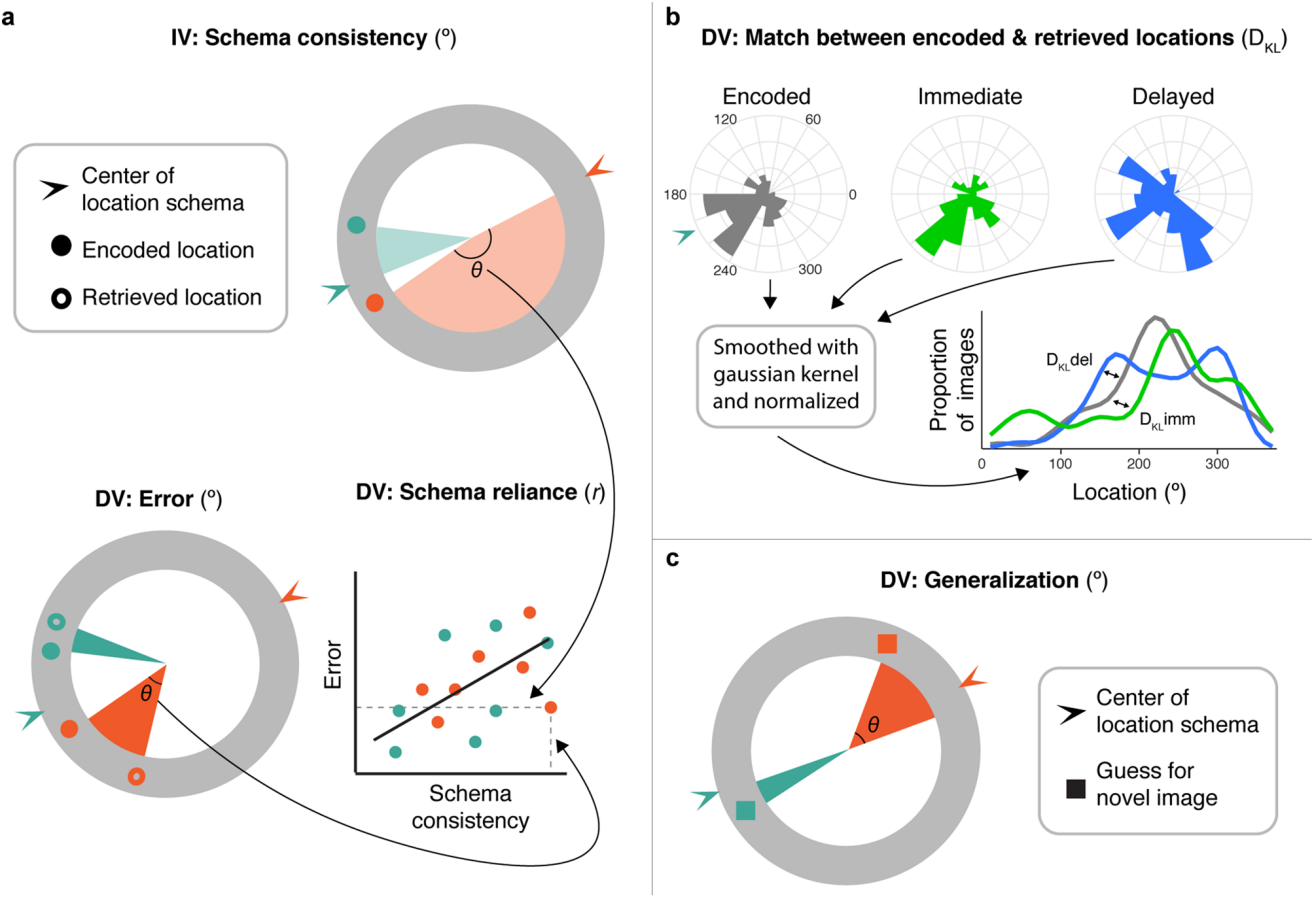
Analysis approach. (**a**) Top: Example retrieval measures for a relatively more schema-inconsistent animal trial (orange) and a relatively more schema-consistent object trial (teal). Schema consistency was operationalized as an image’s angular distance from the center of its category distribution; images ranged continuously in their schema-consistency. Bottom left: Error was defined as the angular distance between an image’s encoded and retrieved locations. Bottom right: the correlation of error and schema-consistency across all trials was used as a subject-level measure of schema reliance. For each subject, this was computed across all tested images, separately for the immediate and delayed tests. (**b**) Subject-level measure of memory for a schema used by Richards and colleagues^7^, operationalized as the Kullback–Leibler divergence (D_KL_) between encoded and retrieved locations, here adapted for use with angle values and separately conducted for animal and object locations. (**c**) Generalization of a schema was defined as a novel image’s angular distance from the center of its category distribution, where lower values signify images placed closer to its category center.

To track episodic and schematic memories separately, we developed novel behavioral measures in addition to adapting ones used in past work. First, we defined an image’s consistency with its category’s ‘location schema’ as a continuous measure, which differs from typical manipulations of schema-consistency. Specifically, an image’s schema-consistency varied continuously as images could be closer to or farther from the center of its category’s spatial distribution (Fig. 3a, top). Continuous-report protocols, commonly used to measure the fidelity of long-term memory^19,20^ and recently employed to demonstrate that the precision of schema-consistent memories is preserved over time^14^, enabled us to track the episodic precision of each association over time (Fig. 3a, bottom left). To assess the use of a schema during episodic retrieval, we integrated error and schema-consistency into a novel, subject-unique measure of ‘schema reliance’ (Fig. 3a, bottom right). To compare these measures with other schema acquisition findings, we adapted a measure used by Richards and colleagues^7^ to infer rodents’ schematic memory for the distribution of platform locations (Fig. 3b). Critically, during the delayed test, participants were also asked to guess where novel images would be located (Fig. 3c). Because these images were never encoded, guesses about their locations can be used to probe the precision of schematic memories while reducing the use of specific episodes.

## Results

### Episodic memory over time

**Figure 4:**
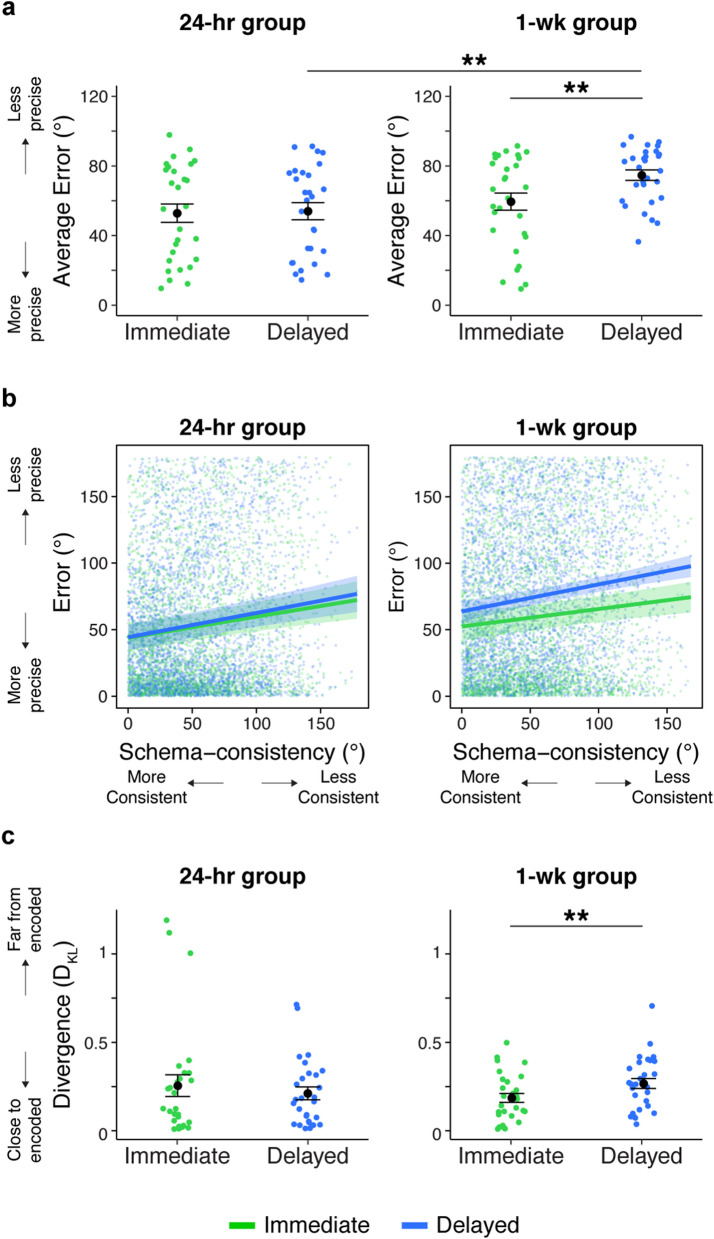
Precision by time and schema-consistency. (**a**) Average error over time. Points represent participants. Error bars signify standard error of the mean (SEM) across participants. Statistics reflect results of trial-level mixed-effects model comparisons. (**b**) The relationship between schema-consistency and episodic precision. Points represent trials. Error ribbons represent 95% confidence interval (CI). (**c**) Divergence between encoded and retrieved locations for each participant. Points represent participants. Error bars signify SEM. **p < 0.01.

Our primary measure of episodic precision for all image-location associations was defined as the angular distance, or error, between an image’s encoded and retrieved location (Fig. 3a, bottom left). Performance was above chance (average error was reliably lower than 90°) at both time points in both the 24-h and 1-week groups (all *t* <  −5.11, all *p* < 0.001, all *d* <  −0.95). We directly compared error in both groups as a function of time in a trial-level mixed effects model with group (24-h, 1-week), time (immediate, delayed retrieval), and their interaction as discrete predictors of error (Supplementary Table 4). We found an effect of group (*t*_(56.93)_ =  −2.23, *p* = 0.03) and of time (*t*_(56.51)_ =  −5.03, *p* < 0.001). These effects were qualified by an interaction between group and time (*t*_(56.51)_ = 4.18, *p* < 0.001). This interaction was driven by increased error at the delayed test relative to the immediate test for the 1-week group (*t*_(58.6)_ =  − 6.47, *p* < 0.001), but not the 24-h group (*t*_(59.3)_ =  − 0.59, *p* = 0.56; Fig. 4a). A direct comparison between groups also revealed increased error in the 1-weeek group relative to the 24-h group after the delayed test (*t*_(59.1)_ =  − 3.61, *p* < 0.001) but not immediately after encoding (*t*_(59.1)_ =  − 0.95, *p* = 0.35). This confirms the expectation that episodic memory was less precise after one week relative to after 24 h. Recognition of the images was also less accurate after one week (see Supplementary Results).

### Relationship between schema consistency and episodic memory

After confirming that the precision of episodic memories decreased over time, we next examined how schema-consistency interacted with this decreased precision, hypothesizing that schematic memory would have a greater influence at a longer delay. Schema-consistency was operationalized as a continuous measure of the angular distance between an item’s encoded location and the center of its category distribution, where smaller values indicate images that are closer to other category members and thus are more consistent with the category’s location schema (Fig. 3a, top). With the model from the prior section that included group (24-h, 1-week), time (immediate, delayed) and their interaction, we added schema-consistency (continuous predictor), and its interaction with group, time, and group by time as fixed effects (Fig. 4b, Supplementary Table 5A). Episodic error, again defined as the angle between the encoded and retrieved locations of each image, was the dependent variable. We found a significant effect of schema-consistency (*t*_(55.46)_ = 7.63, *p* < 0.001), and no interaction between group and schema-consistency (*t*_(55.46)_ = 0.07, *p* = 0.95). This suggests that, when considering both immediate and delayed tests together, schema-consistent images were more precisely retrieved than schema-inconsistent images, and this effect was similar across the two groups. Critically, there was a reliable interaction between time and schema-consistency (*t*_(13099.1)_ =  −2.15, *p* = 0.03), suggesting that the manner in which schema-consistency influenced error differed over time. There was no 3-way interaction between time, schema-consistency and group (*t*_(13099.1)_ = 1.14, *p* = 0.25). Separate plots of error by time and schema-consistency for each participant are shown in Supplementary Fig. 3.

We next computed planned separate models for each group (24-h, 1-week) to understand the interaction between time and schema-consistency (Supplementary Table 5B-C). We focused first on the 1-week group, finding a reliable interaction between time and schema-consistency (*t*_(6713.07)_ =  −2.25, *p* = 0.03) in addition to main effects of schema-consistency (*t*_(28.34)_ = 5.24, *p* < 0.001) and time (*t*_(29.12)_ =  −5.21, *p* < 0.001). While images that were more schema-consistent were more precisely remembered both at the immediate test (*t*_(27.55)_ = 3.83, *p* = 0.001) and delayed test (*t*_(28.76)_ = 4.53, *p* < 0.001), the interaction with time suggests that the influence of schema consistency on error was even stronger at the delayed test. In other words, schemas benefited consistent memories over inconsistent ones at both time points, but after a delay, schema-consistent memories were even more precise than inconsistent ones—despite an overall reduction in precision at the delayed test relative to the immediate one. This suggests that schemas help to preserve consistent memories over time^14,21,22^.

By contrast, in the 24-h group, there was a strong effect of schema-consistency (*t*_(26.92)_ = 5.53, *p* < 0.001) but no main effect of time (*t*_(26.51)_ =  − 1.24, *p* = 0.23) and no interaction between time and schema-consistency (*t*_(6392.95)_ =  − 0.79, *p* = 0.43). There was a positive relationship between schema-consistency and reduced error at both tests (immediate: *t*_(27)_ = 4.93, *p* < 0.001; delayed: *t*_(28.61)_ = 5.01, *p* < 0.001). Thus, although these memories underwent a 24-h period of consolidation, there was no additional benefit of schema-consistency on episodic memory precision at that time period. When considered together with the observations from the 1-week group, it seems that participants in the 1-week group predominantly contributed to the interaction between time and schema-consistency found across both groups. However, because this effect was not reliably different across groups (indicated by the lack of a three-way interaction between time, schema-consistency, and group), it cannot be conclusively claimed that the 1-week group solely contributed to this effect.

Observations from the 1-week group suggest that schemas influence episodic memories more strongly over time, but not in the 24-h group. Interestingly, the overall precision of memories, as measured by the distance between their encoded and retrieved locations, did not decrease after a 24-h delay, suggesting that the influence of a schema may not grow after a delay if the constituent memories giving rise to the schema remain strong. In other words, schematic influence was strongest one week after encoding, when retrieval was less precise overall relative to the immediate test or relative to the 24-h group. This raises an interesting question—are schematic influences on episodic memory time-dependent or strength-dependent? To answer this question, we leveraged the confidence ratings collected during the delayed test. In both groups, high-confident (HC) hits were more precisely retrieved than low-confident (LC) hits and misses (Supplementary Fig. 1A), suggesting that confidence ratings reflected memory strength. We then examined whether confidence modulated the influence of schemas on episodic retrieval. Across both groups, there was a stronger relationship between schema-consistency and error for LC hits and misses relative to HC hits (Supplementary Fig. 1B, Supplementary Fig. 4), mirroring the stronger relationship between schema-consistency and error observed at the delayed test relative to the immediate test.

We also found an intriguing non-linear relationship between average error and schema reliance across individuals. Specifically, participants with moderate average error were more influenced by the location schemas, while participants with the most and least precise memory were less likely to be influenced by the schemas (see Supplementary Results, Supplementary Fig. 2; Supplementary Table 9). This relationship was observed in both groups, at both tests, further highlighting the possibility that the degree of schematic influence may be governed less by the passage of time and more by the strength of the episodes retrieved.

### Memory for encoded distribution

The above results suggest that participants increasingly use knowledge for the learned schemas over time, resulting in more precise memory for images that are most consistent with their category’s location schema. We next wanted to probe memory for the schemas themselves to ask whether it increased over time, adopting an approach used by a recent paper^7^. This approach infers memory for schemas by considering memory for all encoded images as a distribution of locations. We hypothesized that after a delay, participants’ distribution of retrieved locations would more closely match their distribution of encoded locations, despite lower episodic precision, consistent with Richards and colleagues’ observations.

To test this hypothesis, we calculated the divergence (as measured by Kullback–Leibler divergence, Fig. 3b) between each participant’s encoded and retrieved locations, separately for the immediate and delayed tests (Fig. 4c). A 2 (group: 24-h, 1-week) × 2 (time: immediate, delayed) ANOVA revealed no main effect of group (*F*_(1, 55)_ = 0.02, *p* = 0.90, η_p_^2^ = 0.002), no main effect of time (*F*_(1, 55)_ = 0.78, *p* = 0.38, η_p_^2^ = 0.01), and a reliable group by time interaction (*F*_(1, 55)_ = 7.67, *p* = 0.008, η_p_^2^ = 0.12). This interaction was driven by a difference in how the distributions diverged over time across the two groups: participants’ distributions of retrieved locations were less similar to the distribution of encoded locations after a delay in the 1-week group (*t*_(28)_ =  −2.80, *p* = 0.009, d =  −0.57) but not the 24-h group (*t*_(27)_ = 1.26, *p* = 0.22, d = 0.16). One participant in the 1-week group had a relatively high divergence score at the delayed test (although not considered a statistical outlier); after removing this subject, the difference in divergence over time in this group remained statistically significant (*t*_(27)_ =  −2.65, *p* = 0.01, d =  −0.49).

There was no reliable difference in divergence across groups at either test (immediate: *t*_(55)_ = 1.06, *p* = 0.29, d = 0.27; delayed: *t*_(28)_ =  −1.23, *p* = 0.22, d =  −0.33). However, when excluding data from three outlier participants in the 24-h group (> 3 SD from the group mean), participants in the 24-h group diverged from encoding significantly less than participants in the 1-week group during the delayed test (*t*_(52)_ =  −2.69, *p* = 0.01, d =  −0.74). This suggests that the longer the delay, the less participants’ distribution of retrieved locations matched the presented distribution. In summary, participants’ location memory did not grow more similar to the statistical pattern comprising the encoded locations over time; rather, it diverged with this pattern, in contrast to what Richards and colleagues observed (see Discussion).

### Schema generalization: a ‘pure’ test of schema memory

So far, we have demonstrated that participants’ memory is more likely to be influenced by a learned schema in cases when episodic memory strength (measured here as episodic precision) is reduced. This implies that the use of a schema during episodic retrieval depends on whether or not it is needed to bolster memory decisions. However, these observations and, in our read, the related literature in this area adopt a measure of schema memory that may be bolstered by access to individual constituent episodic memories during retrieval. Thus, in order to more directly measure the formation of the schemas, independent from relying on specific episodic memories, we probed if and how participants generalized the schemas to novel images, thus decreasing the need to retrieve specific, precise memory episodes.

**Figure 5:**
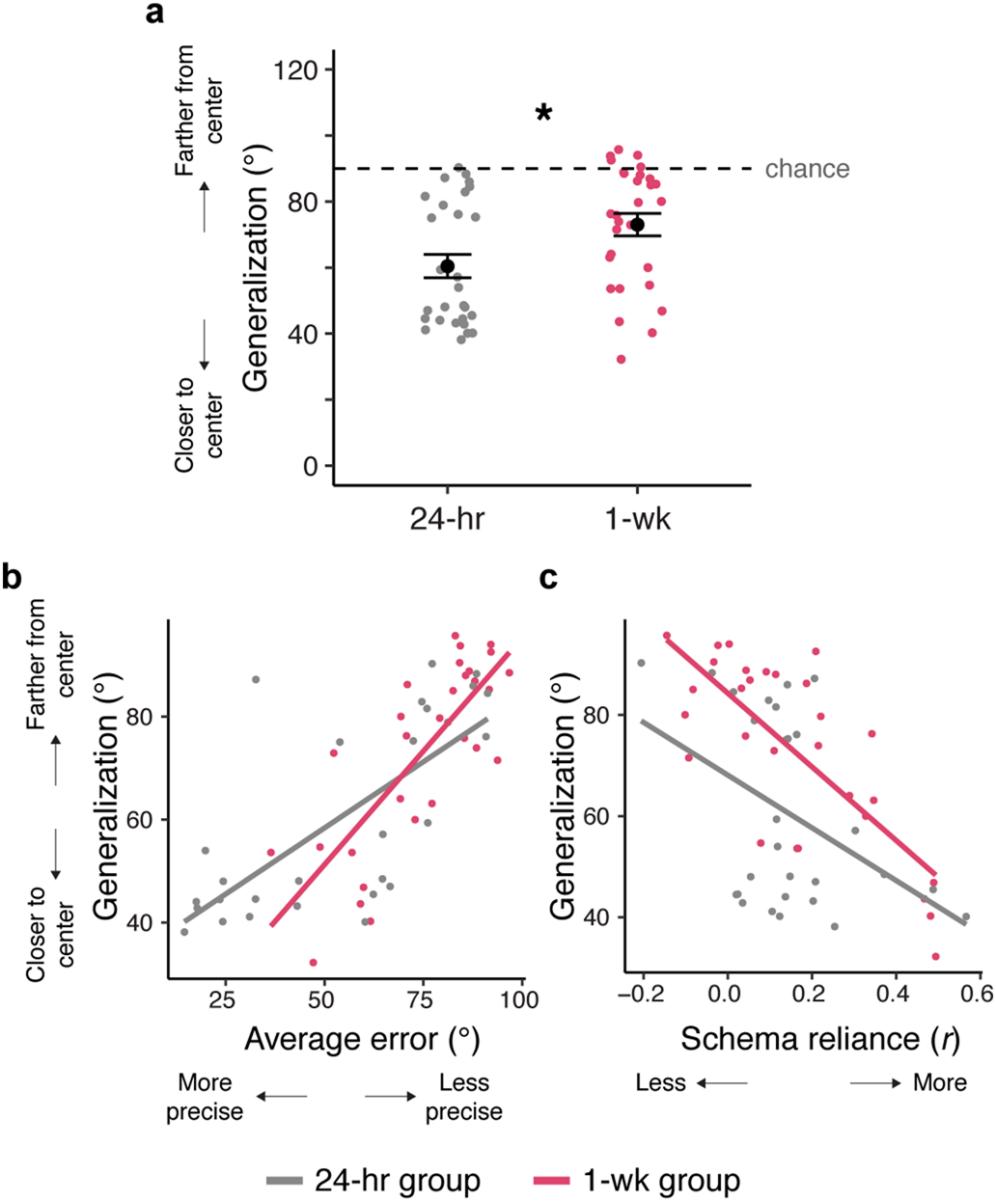
New items. (**a**) Generalization during the delayed test for the 24-h and 1-week groups. (**b**) Correlation between the precision of episodic memories and schema generalization across participants. (**c**) Correlation between schema-reliance during retrieval (correlation between schema-consistency and episodic precision) and schema generalization across participants. Points represent participants. Error bars signify SEM. *p < 0.05.

To this end, novel images were interleaved with old images during the delayed test and participants were instructed to place them on the ring using any information they learned during encoding. Generalization was operationalized as the angular distance between each novel image and its category’s center location, where lower values indicates guesses were closer to the center and thus indicate better generalization (Fig. 3c). Average generalization in both groups was reliably above chance (24-h: *t*_(27)_ =  −8.30, *p* < 0.001, d =  − 1.57; 1-week: *t*_(28)_ =  −5.01, *p* < 0.001, d =  −0.93). Given the observed differences in memory precision between groups at the delayed test, we next asked whether the placement of new images also differed by group, using a mixed-effects model with trial-level generalization as the dependent variable and group (24-h, 1-week) as the independent variable (Supplementary Table 6). Interestingly, we found that participants in the 24-h group placed new images *closer to their category centers* than participants in the 1-week group (*t*_(54.72)_ =  −2.60, *p* = 0.01; Fig. 5a). This suggests that participants’ memory of a schema became less precise over time and shows forgetting of the presented distribution. This observation is especially interesting when considered against the influence of schemas on episodic memory: on one hand, memory for the schemas degraded between 24 h and 1 week after encoding (Fig. 5a), but over the same time frame, they increasingly influenced memory for old images (Fig. 4b, delayed test). This suggests that over time, schemas are increasingly used to support the retrieval of episodic memories despite less precise memory for the schemas themselves.

### Relationship between schema generalization and episodic memory

In the 1-week group, participants’ generalization of a schema was lower than that of the 24-h group, even though their episodic precision was more influenced by schematic memory. This discrepancy led us to wonder whether the manner in which participants retrieved specific episodes was related to their schema generalization. One possibility is that participants with more precise episodic memories would be better equipped to generalize schematic information to new items. Another possibility is that participants who relied most on schemas during episodic retrieval would be better at generalizing the schemas to new images. To differentiate between these two possibilities, we separately correlated participant’s average generalization to new images with two behavioral measures that considered memory for the old images: their average episodic precision and their schema reliance (as measured by the correlation between schema-consistency and episodic precision for each participant, Fig. 3a bottom right).

Focusing first on the relationship between precision and generalization, we computed a linear regression with group and participants’ average error at the delayed test as independent variables and participants’ average generalization (distance between novel images’ locations and their category center) as the dependent measure. This regression found that precision positively correlated with generalization (*β* = 0.69, SE = 0.09, *t* = 7.99, *p* < 0.001) with an effect of group (*β* = 12.75, SE = 6.29, *t* =  −2.03, *p* = 0.048) and a reliable interaction (*β* =  −0.18, SE = 0.08, *t* =  −2.11, *p* = 0.04). In both groups, participants with greater error placed new images further from their category centers (1-week: *r*_(27)_ = 0.78, *p* < 0.001, 24-h: *r*_(27)_ = 0.71, *p* < 0.001; difference in correlations: *z* = 0.55, *p* = 0.58; Fig. 5b), although the significant interaction indicates that schema-consistency explained more variance in precision in the 1-week group. This suggests that participants with more precise episodic memories also exhibited better memory for the corresponding schemas. Alternatively, since the majority of encoded images were schema-consistent (i.e. close to their category center), better memory for these constituents of the schemas may also reflect better learning of schemas themselves. These interpretations are not mutually exclusive, but interestingly, both conflict with the notion that forgetting of details of specific episodes allows for the generalization of information across them^23^.

In a separate regression, we entered group and schema-reliance (each participant’s correlation between schema-consistency and episodic precision) as predictors of new image generalization (Fig. 5c). There was a strong negative correlation between generalization and schema-reliance (*β* =  −62.47, SE = 12.06, *t* =  −2.77, *p* = 0.008), an effect of group (*β* =  −8.10, SE = 2.69, *t* =  − 3.01, *p* = 0.004), and no reliable interaction (*β* = 10.33, SE = 12.06, *t* =  −0.86, *p* = 0.40). In both groups, participants with greater schema reliance placed new images closer to their respective category centers (1-week: *r*_(27)_ =  −0.75, *p* < 0.001; 24-h: *r*_(27)_ =  −0.42, *p* = 0.02; difference in correlations: *z* = 1.87, *p* = 0.06). This suggests that participants with better generalization, or better memory of the schemas, were also more influenced by schemas when retrieving specific episodes.

We next asked whether episodic precision or schema reliance was uniquely associated with the placement of new images. This was important to consider given the non-linear relationship between episodic precision and schema-reliance observed across all participants (Supplementary Fig. 2). To do this, we computed a linear regression with participants’ average generalization as a dependent variable, and three predictors: episodic precision, schema reliance, and group. This revealed an effect of precision (*β* = 0.53, SE = 0.06, *t* = 8.28, *p* < 0.001) and of schema-reliance (*β* =  −49.92, SE = 8.02, *t* =  −6.22, *p* < 0.001), but no reliable effect of group (*β* =  − 1.02, SE = 1.48, *t* =  −0.687, *p* = 0.50). Taken together, these observations demonstrate that both episodic and schema-influenced memory were associated with participants’ generalization of schemas, regardless of whether this generalization task occurred 24 h or 1 week after encoding.

## Discussion

The goal of this study was to better understand both the emergence and expression of schemas over time. To do this, we developed behavioral measures to separately quantify memory for a schema and its influence on the retrieval of specific episodes, and we used these measures to track changes in episodic and schematic memory over time. We found that memory for specific episodes was more precise when the episodes were consistent with a learned schema, and this modulation by schema-consistency was stronger when memories were weaker overall. In other words, schema-consistency more strongly mapped onto variation across memories that were tested after a longer delay and memories that were remembered with low confidence. However, despite this increasing influence of schemas over time, participants’ memory for the schemas themselves declined over time as well, as reflected by the distribution of their retrieved responses and their ability to generalize to novel images. The observations from these measures can be distilled into two conclusions: although memory for new schemas themselves decline over time, they more strongly influence episodic retrieval when those episodes are weakly remembered.

### Schematic influences on episodic memory

We first focused on when schemas are expressed by measuring how they influenced the precision of episodic memories. We investigated this relationship across two factors that reflected differences in episodic memory strength—the delay between encoding and retrieval, and participants’ reported confidence. We found a strong relationship between schema-consistency and precision, where images that were closer to their category center were retrieved more precisely on the immediate test. This relationship became even stronger after 1-week of consolidation, but not after 24-h. This is consistent with observations that schemas facilitate memory retrieval for more consolidated memories^13,21,22^. Moreover, we replicate observations from a similar study, in which participants learned that most images belonging to the same category were located in a certain quadrant of a ring. In that study, the precision of location memories was preserved over 48 h for images whose locations were consistent with their category’s location schema, but decreased for images located in a different quadrant^14^. These findings, however, could be interpreted in one of two ways: schemas were strengthened over time, becoming more influential over episodic retrieval (Fig. 1a), or, as episodes weakened over time, participants increasingly relied on schemas that had formed during encoding (Fig. 1b). Our results provide evidence for the latter interpretation, suggesting that in circumstances where episodic memories were less precise overall (at the delayed test for the 1-week group only), retrieval of each location relied more on schematic memory. Another finding that supports this interpretation is that participants’ reported confidence was also associated with their schema reliance. Specifically, the relationship between schema consistency and error was stronger for low-confident hits relative to high-confident hits both at 24 h and at 1 week. This suggests that reliance on schema information in this experiment was primarily driven by the strength of each episode: weaker memories, either tested 1 week after encoding or remembered with low confidence, were more prone to influence from schemas.

### Schematic memories are forgotten over time

If the expression of a schema is altered based on the strength of the episodes being retrieved, it becomes difficult to disentangle whether schemas become more influential over episodic memory retrieval because they are formed and strengthened over time, or because episodic precision decays over time. To disentangle these possibilities, we developed a generalization test in order to probe memory for the schemas themselves while minimizing the retrieval of specific episodes. We found that this generalization was better in the 24-h group relative to the 1-week group, suggesting that knowledge of the schema memories actually decreased over this time interval. Furthermore, a separate measure of schematic memory used in prior work^7^ paralleled the results of this generalization test. By characterizing memory for a schema as the divergence between participants’ distribution of retrieved locations relative to the locations they had encoded, we found that memories for schemas decayed over time in the 1-week group. However, less precise memory for the schemas was accompanied by an increase in their expression—specifically, relative to the 24-h group, the 1-week group had less precise memory of the schemas, but their episodic precision grew more influenced by the schemas over time. This demonstrates that, over time, increases in the expression of schemas—in other words, the extent to which they influence episodic retrieval—is not necessarily driven by their slow development, but rather by their growing need. The need for schematic memory seems to depend on the strength of episodic memories, and because these memories decay rapidly over time, retrieval is increasingly reliant on schematic rather than episodic memory.

The finding that schema memory decreased over time was surprising, as we had predicted that there would be a stabilization or even improvement in schema knowledge over time. Indeed, there is ample past work in humans showing that time and sleep benefits the extraction and generalization of statistical regularities^10,11,24^, the neural and behavioral integration of overlapping associations^8,9,25^, and new word or grammar learning^18,26–28^. Of particular relevance are observations of enhanced category learning after a delay^19,20^—improvements in the classification of new stimuli over time, using newly learned categories, precisely mirror the learning of a category-location mapping that we have operationalized here as schematic memory. One reason why our findings may conflict with this past work is that the experimental time windows adopted are different. Many studies, in particular those that study the effect of sleep, compare generalization measured directly after encoding and after only one night of sleep, while in our experiment, new images were introduced in both groups one day or one week after encoding. In our study, participants’ ability to generalize may have improved between encoding and the first delayed test, but we did not capture changes in behavior associated with the first night of sleep. In other work that tracked changes in generalization beyond the first night of sleep, participants’ ability to generalize a rule to new stimuli did not improve but rather remained stable^31^. It may be that many time- and sleep-dependent improvements in generalization, in particular for newly created schemas, may not be permanent, but rather emerge after a night of sleep and then decay at a slower rate than memory for episodes. A slower rate of decay for schematic memory would account for the continued and even increasing use of schematic memory to aid the retrieval of specific episodes, and may explain observations that schematic memory persists in the absence of any specific episodes when retrieval is tested at even longer retention intervals^32^.

Another potential reason why schema knowledge decreased over time in our experiment, rather than increasing or remaining stable, is that the experiences that formed the schemas were learned all at once in a compact encoding session. In an experiment in which image-location associations were frequently re-encoded across multiple sessions, recall of the schema-related information remained stable for up to 302 days^33^. Furthermore, our results differ from a similar protocol that directly inspired the development of our own experiment, conducted in rodents studying schema knowledge, or pattern extraction of the locations of platforms in a water maze^7^. In this study, rodents’ swim patterns more closely matched the distribution of platform locations, while crossing fewer specific platform locations, after 30 days relative to 1 day after training. Although we also find that schematic memories increasingly influence episodic retrieval over time, our finding that, when tested separately, schematic memory decays over time, is difficult to reconcile with this result. One reason for the discrepancy with these two past experiments is that participants in our experiment may have been given sufficient encoding trials to learn the location of each image and also extract the pattern of locations by category by the end of encoding. Indeed, if this procedure is thought to be akin to a categorization task, human participants are easily able to learn to classify complex stimuli along multiple dimensions over the course of encoding^34^. Moreover, recent spatial memory experiments in humans show that in a compact encoding session, location schemas can be learned immediately^35,36^ and decay over time^35^, in line with our findings. There is also new evidence in rodents that neural signatures of schematic memories in the prefrontal cortex become activated during encoding, yet only mature with time^37^, suggesting that the groundwork for the extraction of patterns and formation of a schema is present immediately and does not require time to emerge. It could be that, in the absence of a strenuous encoding task, and in cases where encoding is spaced over time, consolidation processes allow for the gradual development of schematic knowledge over time^6^. Indeed, spaced and repeated encoding may capture the emergence of knowledge in a manner that is more ecologically valid than the schemas learned in our experiment. However, such manipulations would be unable to tease apart the acquisition of a schema from its influence on the retrieval of specific episodes, a benefit that only our protocol provides.

### Time-dependent changes in episodic and schematic memory

**Figure 6:**
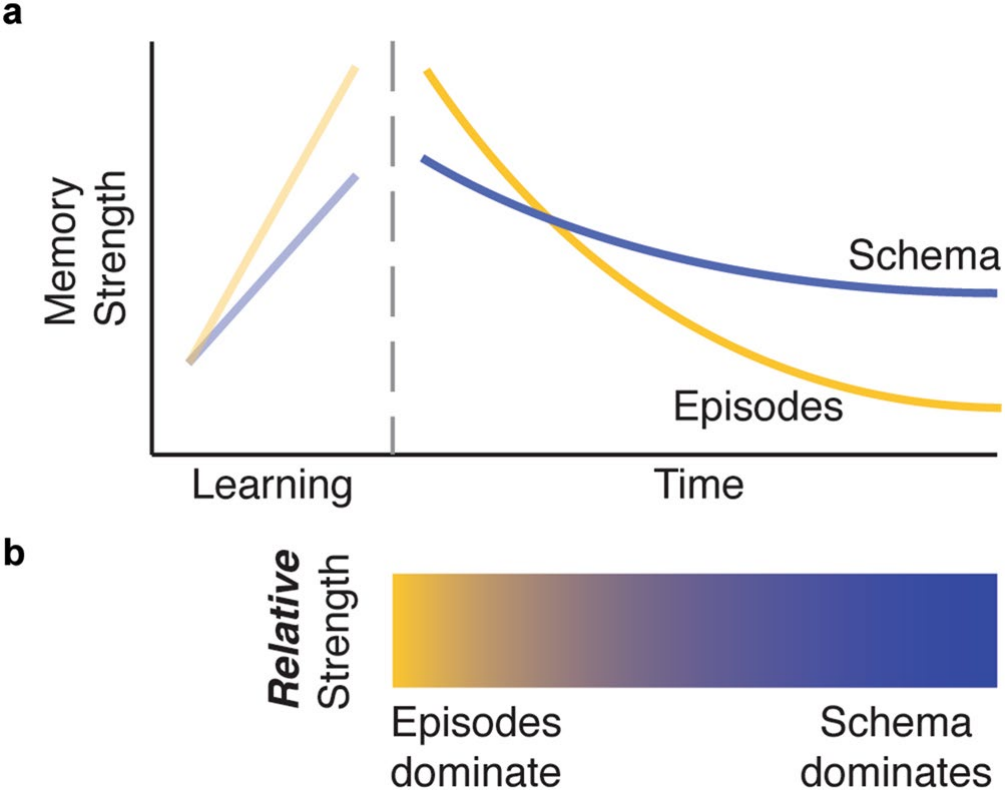
Alternative theory of episodic and schematic memory over time. (**a**) *During learning,* a schematic memory forms concurrently with the episodes that it comprises. *Over time,* it decays, but at a slower rate relative to the decay of its constituent memories. This is different from both hypotheses presented in the introduction, which posited that schematic memory would either grow or remain stable over time while episodes decayed. (**b**) Like the two hypotheses originally presented, retrieval of a schema’s constituent episodes would be more affected by the schema over time, indicated by the yellow-blue gradient.

Taken together, the results reported here suggest that while the precision of schematic memories decreases over time, they nevertheless become increasingly influential in episodic memory retrieval. This pattern of results partially supports the hypothesis that as episodes weaken over time, their retrieval relies more on retained schematic information (Fig. 1b). In contrast to this account, however, we also found that memory for the schemas grew weaker over time, rather than remaining stable. This discrepancy calls for an adjustment, where both episodic and schematic memories are formed during learning, but in the absence of any further stabilization or reinforcement, schemas decay at a slower rate than that of their constituent episodes (Fig. 6a). As mentioned above, a slower rate of decay for schemas over episodes would still result in their increasing influence on episodic retrieval (Fig. 6b). Note that a decrease or decay in memory strength in this framework could be driven by multiple phenomena: forgetting may arise from either decay of the original memory trace or decreasing accessibility to the trace. Our experimental design cannot adjudicate between these two possibilities, but future work may be helpful for understanding the differential trajectories of forgetting in schematic versus episodic memories; for instance, episodic memories may be more susceptible to interference, leading to faster forgetting.

Another way to characterize memory for a schema is to probe how it relates to memory for the episodes that gave rise to it. For example, participants with precise knowledge of the central distribution of a category may also have precise memories of each image’s location. Or, participants whose episodic precision was most influenced by schemas may have better memory for the schemas themselves. To test these two possibilities, we developed separate measures of episodic precision and schema-reliance for each participant and assessed their contribution to the generalization of new items across participants. We found that, at both 24 h and 1 week, good generalization was associated with both high episodic precision and with a stronger reliance on schematic memory. This suggests that the relationship between episodic and schematic memory is bidirectional: while schematic information can aid episodic retrieval, the memory for a schema is also related to memory of its constituent episodes. It further suggests that these two forms of memory develop together, co-exist and can be used interchangeably. Note that even though generalization trials consisted of novel images, generalization of a schema is only an indirect measurement of its strength. It is possible that participants may have relied on memories for encoded images to make their guesses for new images—akin to an exemplar model of categorization^38^—and worse generalization over time may be driven by forgetting of those individual images. Interestingly, this finding contradicts the notion that schematic or general memories are formed through the forgetting of idiosyncratic details of overlapping episodes^23^. We instead find that better memory for the episodes that give rise to a schema are associated with a more precise use of the schema when making decisions about novel information. One interpretation of this finding is that participants who were more engaged in the tasks exhibited more precise memory and better generalization. However, the fact that reliance on schemas during retrieval also was associated with generalization makes this interpretation unlikely. Rather, it suggests that a specific pattern of retrieval errors, guided by schematic information, was related to knowledge of the schema itself. While there are several experimental protocols that measure both the influence of a schema and the memory for its constituent episodic memories^31,39^, there have been few attempts to understand the relationship between the two. Future work is therefore needed to understand the different ways by which schematic information can be extracted from multiple distinct experiences.

### Relationship to other literatures

In our study, we defined a schema as a distribution of locations along a ring at which images of a particular category were likely to be encoded. The majority of encoded images were located near the central tendency of this distribution, with some images encoded farther away. This is different from much of the schema literature in two ways: (1) our measures of schema-consistency and memory precision are continuous, measured by each image’s proximity to the central location of all images of the same category, and the distance between its encoded and retrieved location, respectively, and (2) participants needed to map prior knowledge of category membership onto new, trial-specific spatial information to learn a new schema about the locations of images. Past uses of continuous retrieval reports have enabled researchers to distinguish the precision of episodic memories from their overall retrieval success^40,41^, reveal subtle memory deficits in healthy aging and in patients with altered MTL function^42^, and map separate neural contributions to different aspects of memory retrieval, such as precision, confidence, and vividness^43,44^. In contrast, most research in schematic memory tends to discretize memoranda as schema-consistent or not, using prior knowledge that participants already know, like famous faces, word pairs that are semantically related, or dot patterns that resemble letters^45–47^, although there are numerous exceptions^11,16,17,31^. Despite these differences, we find that images that are closer to their category’s central location are more precisely remembered relative to ones that are farther away, consistent with many past observations that schemas facilitate memory for consistent information^1,47–50^.

The use of a newly formed schema in our experiment bears interesting resemblance to what is known about associative learning. By combining prior knowledge of categories with new memories of image-location associations, participants were able to learn that a general location on the ring was associated with a particular category and use that knowledge to generalize to new images and protect memory for related individual locations. Similarly, there is an abundance of work demonstrating that associative learning can generalize to similar stimuli^51–53^**.** Interestingly, associative learning has been examined at different levels of resolution, suggesting that it can be disentangled into specific, exemplar-based representations that do not generalize and more adaptive associations that may generalize to similar stimuli^54^, a dichotomy that strikingly parallels the dissociation between episodic and schematic memory. Despite these similarities, there seem to be fundamental differences between schema formation and associative learning. First, schemas are thought to be formed from multiple units^3^, which in our case is the building of an association out of a distribution of individual image-location pairs. This basis in multiple episodes is what separates schematic memory from associative learning of one-to-one mappings between pairs or chains of stimuli, like stimulus-stimulus or stimulus-outcome pairings. Furthermore, unlike in traditional associative learning protocols, the category location is not directly trained or reinforced—it is only induced through the combination of participants’ prior knowledge of categories and memory for specific image locations. To date, it is unclear whether the manner in which a more general memory is formed, be it a schema or a broad pattern of associative learning, has consequences for how it is employed. Future empirical comparisons between schema formation and associative learning may better elucidate how differences in their acquisition give rise to similarities in how they guide future behavior.

Our investigations of the relationship between schemas and episodes can be re-framed as a question of how memory is reconstructed out of multiple sources of information^55,56^. In line with a reconstruction account of memory, integration across schematic and episodic information can either enhance or impair memories, depending on whether that information conflicts. In line with this framework, past work has shown that prior knowledge can both enhance and distort episodic retrieval^47,57,58^. In the present study, episodic precision could be affected both by specific episodic memory for an image’s location and by schematic knowledge of the distribution of locations of each category. Integrating across these two sources of information would result in better memory for images that were closer to its category center, since the episodic and schematic memories are providing closely-aligning and reinforcing information. At the same time, memory for an image far from its category’s center would be less precise, because the schematic information is in conflict with the episodic information. Our observation that the precision of memory positively correlates with its proximity to its category center, regardless of the time tested or confidence, provides direct support for this reconstruction account. Another hypothesis that a reconstruction account offers is that memory for images located far from their category center would be biased towards it^14,59,60^. Such a systematic distortion in memory would reflect a strong influence of the schema that is at odds with the true experience. Future experiments designed to capture such biases would provide a complementary approach towards understanding how schemas influence episodic retrieval.

### Limitations and avenues for future work

There are a few limitations to this study to be considered. First, all encoded images belonged to a schema, so we do not have a measure of ‘pure episodic retrieval’ in the absence of schematic influences. A third condition, with images from a third category that were located uniformly around the ring, would be needed to establish a pure baseline measure of episodic precision over time. While not the focus of the current study, without such a baseline, we cannot know whether differences in memory precision by schema-consistency are facilitated or impaired relative to precision in the absence of a schema. In a similar item-location memory experiment^61^ that includes items clustered by a location schema along with items in a no-schema condition, continuous retrieval reports were decomposed into precision and accessibility (the likelihood of retrieving a particular memory rather than guessing). In this study, Berens and colleagues report that memories associated with a schema were more accessible, but less precise, relative to memories in the no-schema condition. At first glance, this finding may seem at odds with our results that schema-consistent memories are more precise than schema-inconsistent ones. However, this apparent discrepancy is well explained by two factors: (1) the use of different precision measures, where ours encompassed all items, while Berens and colleagues calculated precision only from items considered to be true memories, excluding likely guesses, and (2) the notion that retrieval of an item relies both on memory of its category’s location schema and memory of its specific location. When considering both of these factors, items with no related schema (like those presented in Berens et al.) are indeed less likely to be remembered, or less accessible, because they have no schema memory supporting them. However, the memories that are accessible are likely those that are the strongest and therefore the most precise. By contrast, items with an associated schema are more likely to be remembered (more accessible), but with less precision, because the knowledge of the schema additionally supports these memories and thus memory for the specific location need not be as strong. In our data, this reliance on the location schema underlies our finding that schema-consistent images are more precisely remembered than schema-inconsistent ones, highlighting that memory is affected by the distribution of the schema. One test of this interpretation would be to analyze precision for all items from Berens et al.’s schema condition as a function of their distance from the center of their location schema, as we have done in the current analyses. When precision is computed over all trials, including likely guesses, we predict that memory for schema-consistent items would be more precise than that of no-schema items, as these memories would have the additional support of the related schema such that both strong memories and guesses would result in retrieval close to their encoded location. In contrast, memory for schema-inconsistent items would be less precise than no-schema items, because guesses for schema-inconsistent items would be in conflict with memory for the related schema, leading to distortion^60^. A second limitation is that, when possible, we tested for within-subject relationships between schema-consistency and episodic precision, and we also were able to leverage differences between the immediate and delayed tests within each group as within-participant controls. However, many of our critical comparisons of performance, in particular comparisons of schema generalization, were computed using between-subjects analyses. Because of this, any behavioral differences between these conditions may be driven in part by variance across participants, rather than differences in memory over time. Note, however, that the performance of these two groups at the immediate test was matched in various ways: their episodic precision did not reliably differ, the divergence of their remembered locations from the distribution that generated their encoded locations did not differ, and neither group exhibited a reliable relationship between schema-reliance and average error across participants. While these between-subjects analyses are useful, future work will be better equipped to quantify differences in forgetting of episodic and schematic memory using a fully within-subject design.

It is also important to note that our measure of generalization to new images was developed to minimize the use of specific episodes when assessing the precision of knowledge for the location schemas. However, it is likely that participants’ memory for specific image locations still informed their performance on this task—for example, participants may have placed a new image of a tiger at the location that a lion was encoded. Although we did not systematically vary the semantic similarity of category members, which would have allowed us to test this possibility, we did observe a correlation between episodic precision and generalization, suggesting that less precise memory for episodes would explain less precise memory for the schemas over time. However, the observation that generalization decreased over time is still interesting to consider in light of the observation that participants’ memory for encoded images was increasingly influenced by their schema knowledge after a delay. If it is the case that both episodic precision and schematic knowledge decreased over time, the notion that participants increasingly relied on schemas over the same time interval suggests a more complex relationship between the two sources of memory. Since schema knowledge by definition is built by accumulating information across separate episodes, it is likely that no behavioral measure is capable of assessing the acquisition of schema knowledge in the complete absence of memory for its episodes—thus, we aimed to minimize the influence of specific episodes, rather than completely eradicate it. Another disadvantage of the generalization measure was that it assumed participants’ schema knowledge was reflected by their estimate of the center of each category’s location schema, such that images placed closer from the center was interpreted as better schema knowledge. An alternative explanation is that participants who happened to be more variable in their responses, perhaps due to fatigue, placed images anywhere in the category’s section but not necessarily close to its center. If so, our measure fails to capture the extent of their schema knowledge. Future work, perhaps with more sophisticated approaches for measuring schema knowledge, may be able to isolate the bidirectional interactions between schema knowledge and episodic memory.

In summary, we provide evidence that the formation of a schema can be disentangled from its expression, demonstrating that schemas are increasingly used as episodic memory decays over time, even as precise knowledge of the schemas decays. We provide a comparison of various behavioral measures used to track memory for specific episodes and assess knowledge of the schemas that emerge through their integration, with an eye towards improving the analytical tools to disentangle the acquisition and transformation of these forms of memory in future work. Finally, we propose that considering the relative strengths of schematic and episodic memory provides testable predictions about how this information is integrated to support retrieval.

## Methods

### Participants

Participants were recruited from the New York University (NYU) subject database and were compensated with class credit or money. 29 subjects (9 males) participated in the 1-week group and 28 (7 males) in the 24-h group. Eligible subjects were: (a) between 18 and 30 years old, (b) right-handed, (c) native English speakers or English speaking for 10 years, (d) normal or corrected to normal vision and (e) normal color vision. Subjects provided written Informed Consent at the beginning of the study. The research was approved by the University Committee of Activities Involving Human Subjects (UCAIHB) at New York University. All methods were carried out in accordance with relevant guidelines and regulations.

### Stimuli

#### Images

168 color images of everyday objects were used in this study. The images were selected either from Hemera Photo-Objects 10000 Premium Image Collection (http://www.bmsoftware.co.uk/hemeraphotoobjects10000.htm) or from Google Image searches. Images were cropped to 400 × 600 pixels such that each object was centered and occupied a consistent proportion of the image. For each subject, the images were randomly divided into two sets: 120 for encoding, and 48 for novel foils in the delayed test.

#### Locations

For each subject, the center of one category’s distribution was randomly chosen within 0 to 2π radians. Angles within 0.0873 radians (5°) of the vertical and horizontal axes were excluded. The second category’s center was located π radians away (on the opposite side of the ring). Then, for each category, 60 values were randomly drawn from a cosine distribution centered around the chosen angle:

$$f\left( x \right) = \frac{{\cos \left( {x - center} \right) + 1}}{2}$$

These randomly drawn values became the angular locations along the ring, where *θ* = 0 pointed to the right and *θ* = π/2 pointed to the top of the screen. For each category, the resulting 60 locations were randomly paired with the 60 images chosen for encoding.

The use of cosine functions ensured that when collapsing across category, the distribution of all 120 locations was roughly uniform and participants could not learn to bias their mouse movements towards particular sections of the ring. Separate chi squared tests for each participant confirmed that the overall distribution of images was not reliably different from a uniform distribution in both experiments (all χ^2^ < 15.50, all *p* > 0.07).

#### Software

Stimulus presentation code was written in Matlab 2014 using the Psychophysics Toolbox extension^62^. All analyses and statistical tests were conducted using R^63^.

### Procedure

The experiment took place in a soundproof testing room. The experiment consisted of two sessions, with Session 2 taking place one week after Session 1 in the 1-week group and 24 h after Session 1 in the 24-h group (Fig. 2a). All other aspects of the design are identical across the two groups. In Session 1, subjects completed encoding and immediate retrieval. In Session 2, subjects completed delayed retrieval. Instructions for each task were given right before the task both verbally by the experimenter and in written form on the screen.

#### Encoding

Subjects were instructed to learn the association between each image and its location along the ring. They were told that their memory for the images' locations would be tested later in the experiment. Encoding was divided into six 60-trial blocks, with blocks 1–2, 3–4, and 5–6 forming three cycles of stimulus presentation. Within each learning cycle, all image-location pairs were presented once in random order. All pairs were thus viewed 3 times. Each block was separated by a one-minute break.

On each trial (Fig. 2b), an image was presented at the center of the screen for 2 s. Then, a ring with a radius of 400 pixels and width of 40 pixels appeared with a smaller version of the image (4% of its original size) at its center. The location paired with the image was marked on the ring by a red line. Subjects were asked to move the image to the red line and click the left mouse button when the image was at the target location. The mouse cursor was linked to the center of the image such that any mouse movement moved the image. To ensure that participants were engaged during the encoding task, clicks for images that were 40 pixels or closer to the target location were interpreted as valid responses. All others would trigger a warning sound that prompted the subjects to move the image closer to its location. After a valid response, or if no valid response was made within 3 s, the image would appear at its associated location. The image remained at its location for 2 or more seconds depending on the speed of the response, so that the duration of all trials was matched at 7 s. Trials were separated by a 0.5 s fixation period. The mouse position was reset to the center of the ring at the beginning of the subsequent trial.

#### Immediate retrieval

Directly after completing the encoding phase, participants completed the immediate retrieval test (Fig. 2c). In this test, subjects were asked to retrieve the locations associated with the presented images and instructed to guess if they could not remember the correct location. They were also instructed to use the provided feedback as a final opportunity to learn the image-location pairs in preparation for a future memory test. All 120 image-location pairs were tested in random order, divided into two 60-trial blocks separated by a one-minute break.

The presentation, timing, and mouse movements in this task were identical to the parameters from encoding, with the exception that the red line marking the location of each image was not shown. Any click on the ring was categorized as a valid response. Mouse clicks when the image was not on the ring would trigger a warning sound to prompt the subjects to move the image onto the ring. If a valid response was made, or if no response was made within 3 s, the image would appear at the location paired with it. This feedback was included in order to minimize potential retrieval-induced error in the delayed test. Importantly, immediate retrieval differed from delayed retrieval in that no novel images were shown (see below); this was to minimize participants’ attention to the location schemas and reduce the influence of this knowledge on the consolidation of memories of specific item-location associations.

#### Delayed retrieval

During the delayed retrieval test (Fig. 2d), participants viewed all 120 pairs interleaved with the 48 novel foils. The order of presentation was pseudo-randomized so that no more than two consecutive trials were of foils. Subjects were asked to retrieve the locations associated with the encoded images and were instructed to guess if they could not remember. For foils, they were asked to make their best guess about what its location could be, using any information they had learned during the encoding task. The 168 trials were divided into three 56-trial blocks separated by 1-min breaks. The presentation, timing, mouse cursor, and warning sounds in this task were identical to the parameters from immediate retrieval, with the exception that images were not presented at their correct locations after a response was made.

Instead, after each response was recorded, participants rated their confidence for the location they had chosen with a 4-point scale. Options ranged from 4 (*“very sure”*) to 1 (*“not sure”*). If participants considered the image to be a foil, they were instructed not to use the confidence scale and instead press “new”. The five options were mapped to “z”, “x”, “c”, “v” and “b”, and the mapping was counterbalanced between subjects. Once a rating was chosen, or if no rating was made within 3 s, the scale would disappear and the trial ended. Trials were separated by a 0.5 s fixation period.

### Analysis

#### Error

The precision of memory for the locations associated with each image was evaluated by quantifying the magnitude of error for each trial. Error was defined as the absolute value of the angle between an image’s encoded location and its retrieved location, with reference to the center of the ring. The error for a given image ranged from 0 to 180 degrees, where smaller values indicate more precise memory retrieval. Trial-specific error values were either entered into mixed-effects models, or averaged across trials by retrieval test or confidence for across-participant correlations and visualization of mixed-effects models. Comparisons to chance performance were conducted with two-tailed one-sample t-tests of participants’ average error against 90 degrees, which is the average error that reflects random guessing.

#### Schema-consistency

Schema-consistency was operationalized as the consistency between an image’s location and the ‘location schema’ that could be learned for each category. This was computed as the distance between each image’s encoded location and the center of the distribution of locations corresponding to the image’s category. In mixed-effects models predicting error, schema-consistency was entered as a continuous, fixed-effects independent variable.

#### Generalization

Generalization of a schema was operationalized using the locations of new images. For each trial, generalization was defined as the angular distance between each image and its corresponding category’s center location. The generalization for a given image could range from 0 to 180 degrees, where lower values indicate guesses that are closer to the center of an image’s category and thus better generalization. Comparisons to chance performance were conducted with a two-tailed, one-sample t-test of participants’ average generalization against 90 degrees, which is the average distance from a category center that reflects random guessing. The comparison of generalization across groups was conducted with a mixed-effects model using group (1-week, 24-h) as a discrete fixed effect and generalization as a continuous dependent variable.

#### Divergence analyses

To calculate the Kullback–Leibler divergence (D_KL_) between the distributions of locations in different tasks, the image locations of each task (encoding, immediate retrieval, and delayed retrieval) were converted into probability density functions to be comparable with the distributions used to generate encoded locations for each category. For each task, the locations were first divided into 36 10-degree bins of the ring. The resulting distribution of locations per bin were normalized to sum to 1, resulting in a probability density function of the locations around the ring. Each function was smoothed by convolution with a Gaussian distribution (σ = 22°) in order to avoid bins with no locations, which would lead to extreme D_KL_ values. While the choice of σ was not motivated by prior work, the specific σ was not critical as using different values produced similar results. For any two distributions, the D_KL_ was calculated with the following equation:

$$D_{KL} (P_{Encoded} || P_{Retrieved} ) = \mathop \sum \limits_{x = 1}^{37} P_{Encoded} \left( x \right)\ln \left( {\frac{{P_{Encoded} \left( x \right)}}{{ P_{Retrieved} \left( x \right)}}} \right)$$

D_KL_ was computed to compare the distribution of encoded images with the retrieval distributions separately for the immediate and delayed retrieval tests. Greater D_KL_ indicates a larger mismatch in the distribution of images between two tasks.

Three participants in the 24-h group were statistical outliers (> 3 SD from the group mean), and one participant in the 1-week group exhibited a relatively high divergence score at the delayed test (based on visual inspection). To account for these outlier participants, we report the differences in divergence over time and across groups with and without these participants included. Furthermore, these participants were not statistical outliers in any other analysis reported in this manuscript, and excluding them does not meaningfully change the observed results.

#### Mixed-effects models

Because participants were given a limited amount of time to encode and retrieve each trial, the number of completed trials per participant varied (See Supplementary Tables 1, 2 and 3 for response rates by task, group, and confidence). Because of this, all analyses were conducted at the trial level with mixed-effects linear models, except for across-participant correlational analyses and comparisons against chance performance. This included analyses of the effects of schema-consistency, time and confidence on error (Fig. 4a, b, S1A, B) and group differences in generalization (Fig. 5a). When needed, model convergence warnings were avoided by scaling error and schema-consistency across all trials and participants to be centered around 0 with SD = 1. Participant intercepts and slope terms for each included predictor variable were modeled as random effects. The mixed-effects models were computed with *lme4*^64^ and significance of a given contrast was obtained using Satterthwaite’s method, resulting in *t* statistics and corresponding *p* values. Analysis of variance was conducted for models containing discrete predictors with more than two levels (i.e. Supplementary Fig. 1A, Supplementary Fig. 2) using the *lmerTest* package^65^. Estimated marginal means (EMMs) were computed using the *emmeans* package^66^ to test simple effects. To facilitate visualization and interpretation of the results, all data and model fits were un-scaled, and analyses with discrete independent variables predicting error (Figs. 4a, 5a, S1A) were plotted as error averaged within each participant.

#### Across-participant correlations with generalization

Generalization to the new images was used to index variations in participants’ schema knowledge. This was operationalized as the mean of the distances between all new images’ guessed locations and their category center. Lower values indicate closer placement of images to their ‘category schema’. Linear regressions were used to compute across-participant relationships between generalization, average error and schema-reliance. Pearson correlations were used to quantify the relationship between generalization and average error or schema-consistency for specific groups and time points.

#### Effect sizes

Effect sizes were reported for all effects, including Cohen’s *d* for all t-tests and partial η^2^ for main effects or interactions of ANOVAs, computed with *sjstats*^67^. Tables of the estimates and standard error of the fixed and random effects of each reported model can be found in the supplementary materials.

## Supplementary information

Supplementary information.

## Data Availability

All data and analysis code that support the findings of this study are available at https://osf.io/t2bez.
