# Supplementary material for: Schematic memories develop quickly, but are not expressed unless necessary
Source: Sci Rep. 2020 Oct 12;10:16968. doi: 10.1038/s41598-020-73952-x (PMC7550328; doi:10.1038/s41598-020-73952-x)
Supplement: Supplementary file 1 — Supplementary information. [file 41598_2020_73952_MOESM1_ESM.pdf]

Schematic memories develop quickly, but are not expressed unless necessary

Alexa Tompary[1]*, WenXi Zhou[2], Lila Davachi[3,4]

[1]Department of Psychology, University of Pennsylvania, Philadelphia, PA 19104
[2]Center for Neural Science, New York University, New York, NY 10003
[3]Department of Psychology, Columbia University, New York, NY 10027
[4]Nathan Kline Institute for Psychiatric Research, Orangeburg, NY, 10962

*Corresponding author:
Alexa Tompary
atompary@sas.upenn.edu

Supplementary Text:
21 pages, 4 figures, 9 tables

Supplementary Text

Completed trials: Participants were given 3 seconds to move each image to its associated location during encoding and retrieval. If participants did not respond in time, the experiment automatically advanced to the next trial. Responses in the second and third rounds of encoding were not recorded for one participant in the 1-week group due to experimental error. The average proportion of completed trials for each group and task are listed in Supplementary Table 1 (encoding) and Supplementary Table 2 (retrieval).

Recognition: The confidence measure was also designed to assess recognition accuracy at the delayed test. For this analysis, high- and low-confident correct trials were collapsed into one bin to represent all hits, and novel foils were divided into false alarms and correct rejections. Recognition was indexed with A[1], a non-parametric measure of sensitivity which ranges from 0 to 1, with 1 indicating perfect sensitivity (100% hits and no false alarms). Participants exhibited high recognition for the images in the 1-wk group (mean A = 0.77, SD = 0.14) and in the 24-hr group (mean A = 0.85, SD = 0.16). As expected, recognition was significantly better in the 24-hour group relative to the 1-week group ($t_{(55)}$ = 2.07, p = 0.04, d = 0.55).

Confidence ratings: As part of the recognition test, participants in the two groups rated confidence for their location memory. Specifically, participants rated how confident they were about their retrieval of each image's spatial location on a 1 to 4 scale, with an additional option of 'New' if they did not recognize the image. Responses were sorted into three conditions based on participants' responses: high confident hits, with a confidence rating of 3 or 4; low confident hits, with a confidence rating of 1 or 2; and misses, which were endorsed as 'new'.

We tested whether participants' proportion of responses to old images differed with a 2 (group: 24-hr, 1-wk) by 3 (response: HC hit, LC hit, miss) ANOVA. This revealed a main effect of response ($F_{(2,110)}$ = 34.67, p < 0.001, η_p^2 = 0.39), no main effect of group ($F_{(1,55)}$ = 1.04, p = 0.31, η_p^2 = 0.00), and a group by response interaction ($F_{(2, 110)}$ = 4.08, p = 0.02, η_p^2 = 0.07). To understand this interaction, we compared the

proportion of each response across groups. Participants in the 24-hr group responded with more HC hits than participants in the 24-hr group ($t_{(55)}$ = 2.26, p = 0.03, d = 0.60), and but there were no reliable group differences in the rate of LC hits ($t_{(55)}$ = -1.75, p = 0.09, d = -0.46) or misses ($t_{(55)}$ = -1.71, p = 0.09, d = -0.45). Average response rates for each group are reported in Supplementary Table 3.

Episodic precision by confidence: To assess whether confidence modulated episodic precision, we entered average error in the delayed test into a mixed-effects model with group (24-hr, 1-wk) and confidence (HC hit, LC hit, miss) as discrete predictors (Supplementary Figure 1A, Supplementary Table 7). An analysis of variance revealed main effects of group ($F_{(1, 54.2)}$ = 9.93, p = 0.003) and confidence ($F_{(2, 104.6)}$ = 48.39, p < 0.001), and no reliable interaction ($F_{(2, 104.6)}$ = 2.15, p = 0.12). In both groups, HC hits were more precise than LC hits (24-hr: $t_{(38.5)}$ = -7.90, p < 0.001; 1-wk: $t_{(42.1)}$ = -6.48, p < 0.001) and missed trials (24-hr: $t_{(56.4)}$ = -5.94, p < 0.001; 1-wk: $t_{(37.2)}$ = -3.71, p < 0.001). Precision of LC hits was not reliably different from missed trials in either group (24-hr: $t_{(47.9)}$ = -1.09, p = 0.28; 1-wk: $t_{(37.4)}$ = 1.26, p = 0.22). When comparing across groups, error did not differ for missed trials ($t_{(46.3)}$ = -1.80, p = 0.08), but the 1-week group exhibited greater error relative to the 24-hour group for both HC hits ($t_{(56.8)}$ = -2.97, p = 0.004) and LC hits ($t_{(57.6)}$ = -3.55, p < 0.001). Together these results confirm that participants' confidence ratings were associated with error in their location memory, and thus are a reliable indicator of episodic precision.

***Relationship between confidence and schema-consistency**:* After establishing that confidence was associated with error, we next asked whether confidence interacted with schema-consistency to modulate error (Supplementary Figure 1B). We computed a mixed-effects model with group, confidence, schema-consistency, and their interactions as fixed effects and error as the dependent measure (Supplementary Table 8). We collapsed LC hits and misses into one bin as there was no reliable difference in precision for these confidence responses in either group. Critically, there was a confidence by schema-consistency interaction ($t_{(3755.81)}$ = -3.21, p = 0.001), revealing that confidence modulated the relationship between schema-consistency and error. This

model also revealed an effect of schema-consistency ($t_{(57.96)}$ = 6.71, p < 0.001) and no schema-consistency by group interaction ($t_{(58.0)}$ = -0.07, p = 0.94). There was no reliable three-way interaction ($t_{(1,\ 3755.81)}$ = -1.7, p = 0.09), suggesting that this relationship between confidence and schema-consistency did not reliably differ across the two groups. Separate plots of error by confidence and schema-consistency for each participant are shown in Supplementary Figure 4.

For an analogous comparison to our analyses of schema-consistency over time, we next entered confidence and schema-consistency into separate models for each group. When focusing on the 1-week group, a model that included confidence, schema-consistency, and their interaction revealed main effects of schema-consistency ($t_{(30.23)}$ = 4.33, p < 0.001) and confidence ($t_{(26.35)}$ = -5.50, p < 0.001) but no reliable interaction ($t_{(2204.52)}$ = -1.07, p = 0.28). In the 24-hour group, we again found strong main effects of schema-consistency ($t_{(27.77)}$ = 5.46, p < 0.001) and confidence ($t_{(25.61)}$ = 61.64, p < 0.001). However, in contrast to the 1-week group, this model revealed a reliable interaction between schema-consistency and confidence ($t_{(1376.1)}$ = -7.85, p < 0.001), where after a shorter retention interval, schema-consistency was more strongly correlated with error for LC hits than for HC hits. In contrast, after 1 week, confidence did not modulate the relationship between schema-consistency and error. One possible reason for a lack of an interaction in the 1-wk group may be that participants changed their criteria for rating confidence after a longer delay, such that HC hits across the two groups reflected memories of different strengths. As an example, participants' rates of confidence responses differed across the 24-hr and 1-week groups, where the 1-week group endorsed fewer responses with high confidence than the 24-hr group (See Supplementary Text, Supplementary Table 3).

In the 24-hour group, HC hits were less likely to be modulated by schema-consistency relative to LC hits. Interestingly, this finding mirrors the observed difference in how schema-consistency influenced precision over time in the 1-week group. The fact that similar effects were observed within the same retrieval test, rather than across time, suggests that the likelihood that a schema will benefit memory does not so much depend on the age of the memory, but rather on its strength.

Relationship between episodic memory and schema reliance across participants: The results reported in Figure 4 suggest that schemas influence episodic memory within individuals: schema-consistent memories were more precisely retrieved relative to inconsistent ones, and that this difference was magnified after a delay, after episodic memories became less precise overall. If schematic memories provide additional support for episodic retrieval, then it may also be the case that participants who relied more on schematic memory may have better episodic memory. To test this, we developed a measure of schema reliance for each participant, by computing the correlation between error and schema-consistency across trials separately for each participant. In other words, a participant with higher schema-reliance would have more precise memory for schema-consistent memories relative to inconsistent memories, whereas a participant with no schema-reliance would have similar precision for schema-consistent and -inconsistent memories. We correlated this measure with each participant's average error across all trials.

To match this analysis as closely as possible to the trial-level analyses reported in the main text, we began with a mixed-effects model with average error, group (1-week, 24-hour), time (immediate, delayed), and their interactions as fixed effects and schema-reliance as the dependent variable. There were two observations per participant in the model (one per retrieval test). Participant intercepts were modeled as random effects with no random slope terms. We compared this model against one that included a quadratic term to explain error and its interactions with time and group (Supplementary Figure 2; Supplementary Table 9), finding that the quadratic model was a significantly better fit relative to the linear model ($\chi^2(4) = 18.65$, p < 0.001).

An analysis of variance of this quadratic model revealed a significant effect of error ($F_{(2, 94.9)} = 15.10$, p < 0.001). There were no other reliable effects of time, group, or their interactions with error (all $F < 0.94$, all $p > 0.33$). Across both groups and both tests, participants with moderate episodic precision were most influenced by the schemas, while participants with the most precise memory exhibited less of an influence, as did participants with precision approaching chance levels (errors around 90°). These results provide complementary evidence that the influence of schema knowledge is best explained by memory strength, rather than the passage of time.

References

1. Zhang, J. & Mueller, S. T. A note on ROC analysis and non-parametric estimate of sensitivity. *Psychometrika* **70**, 203–212 (2005).
2. Richards, B. A. *et al.* Patterns across multiple memories are identified over time. *Nat. Neurosci.* **17**, 981–986 (2014).

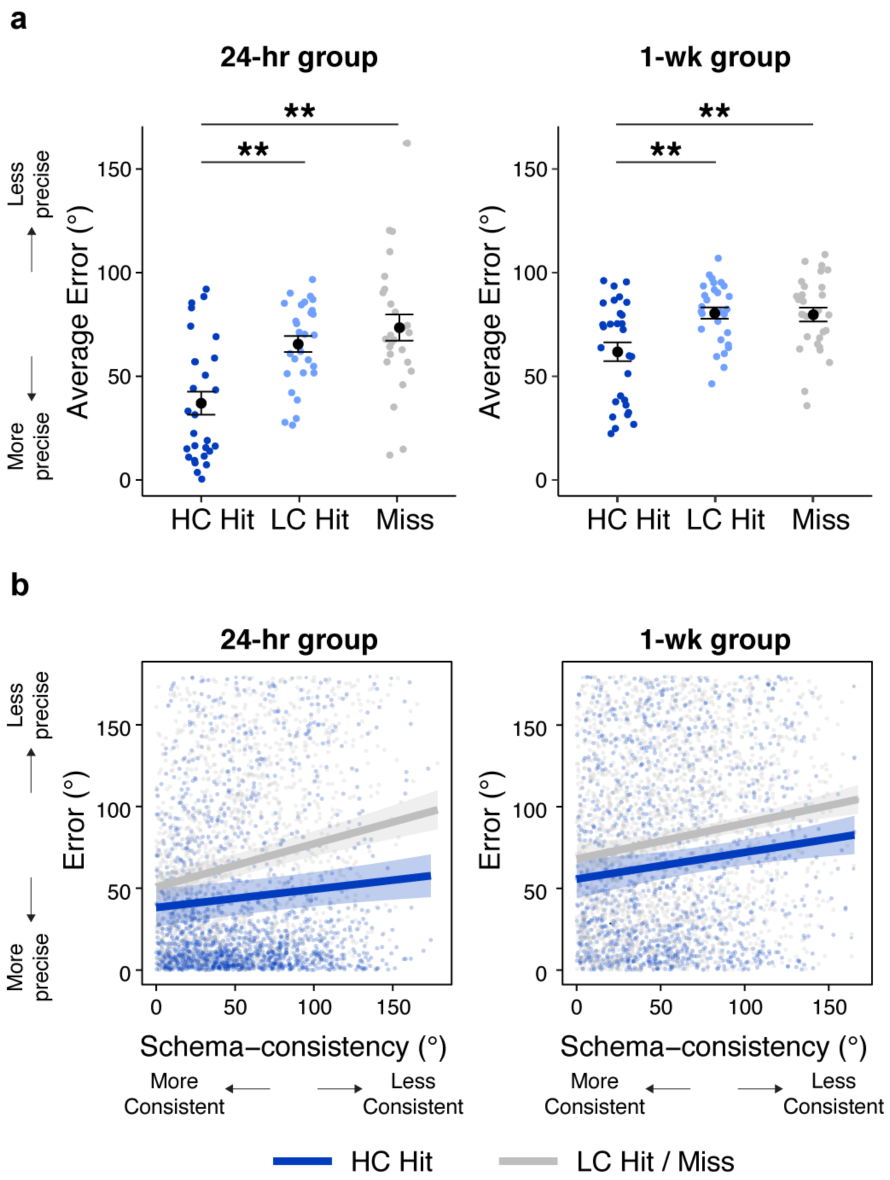

Supplementary Figure 1. Precision by confidence and schema-consistency. **(A)** Average error at the delayed test by confidence. Points represent participants. Error bars signify SEM. Statistics reflect results of trial-level mixed-effects model comparisons. **(B)** Mixed effects model of the effects of confidence and schema-consistency on error. Points represent trials. Error represents 95% CI.

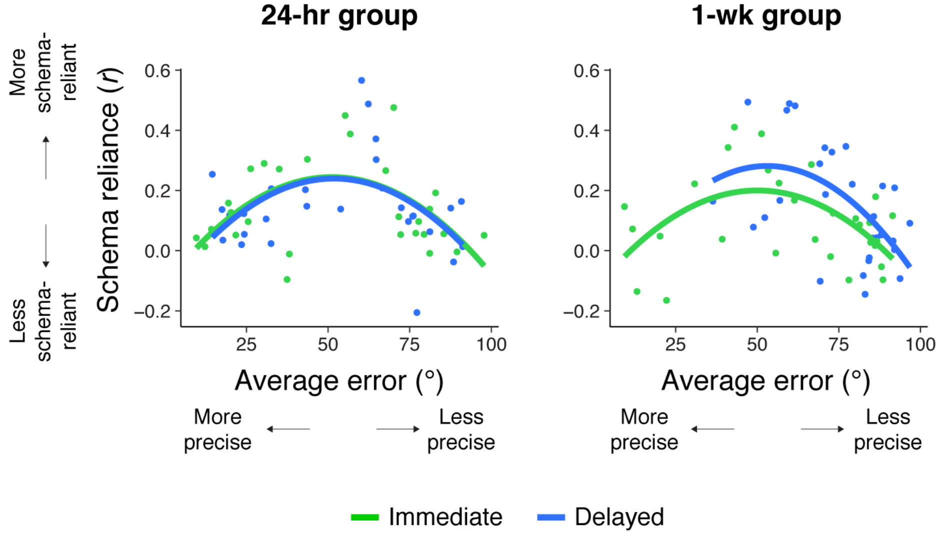

Supplementary Figure 2. Across-participant correlations between average episodic precision and schema reliance. Schema reliance was operationalized as the correlation between schema-consistency and precision within participants, where larger values indicate a greater difference in precision for schema-consistent relative to schema-inconsistent trials. Points represent participants.

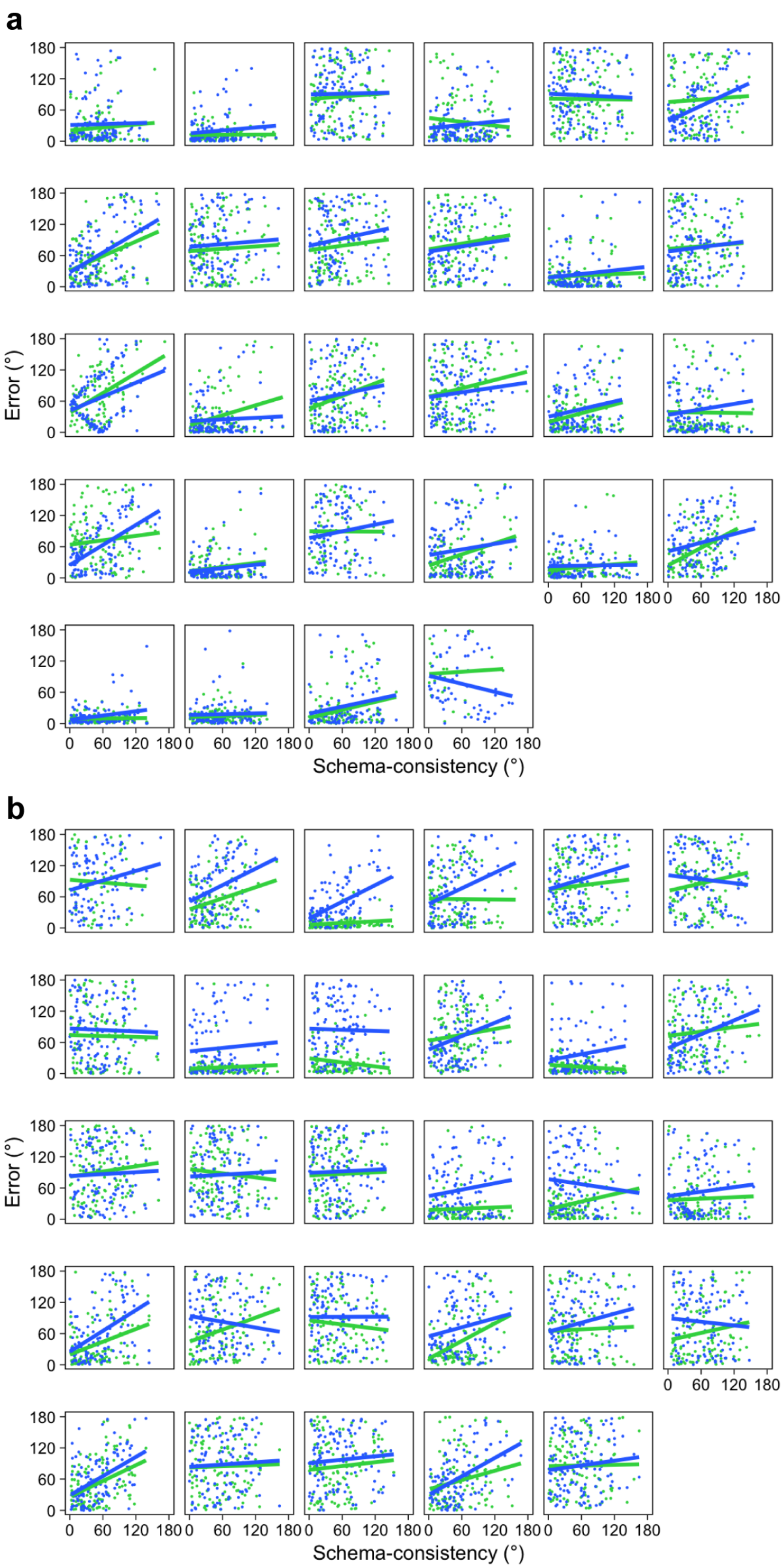

Supplementary Figure 3. Separate plots of error as a function of schema-consistency and time for each participant. Points represent trials. Green indicates immediate test; blue indicates delayed test. **(A)** 24-hour group. **(B)** 1-week group.

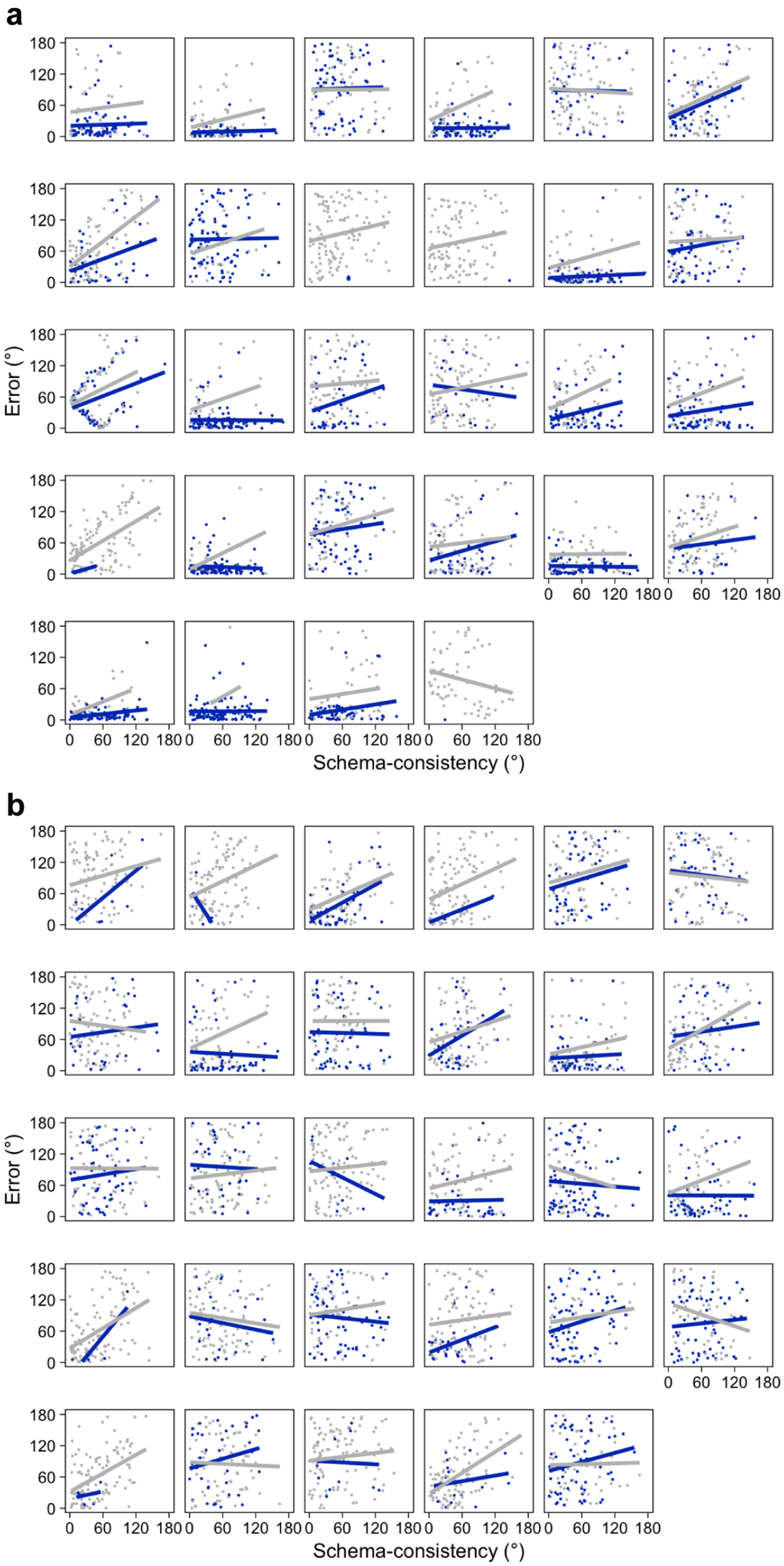

Supplementary Figure 4. Separate plots of error as a function of schema-consistency and confidence for each participant. Points represent trials. Dark blue indicates HC hits; gray indicates LC hits and misses. **(A)** 24-hour group. **(B)** 1-week group.

Round	24-hr group	1-wk group
First	92.6% (15.3%)	96.5% (9.5%)
Second	94.0% (16.9%)	96.3% (12.5%)
Third	94.7% (17.5%)	96.8% (7.1%)

Supplementary Table 1. Mean and standard deviation of proportions of completed trials during the three rounds of encoding.

**Response**	**Trial type**	**24-hr group**	**1-wk group**
Immediate	Old	94.3% (16.7%)	95.9% (10.1%)
Delayed	Old	96.9% (10.8%)	99.0% (2.6%)
Delayed	New	97.1% (9.0%)	98.5% (2.4%)

Supplementary Table 2. Mean and standard deviation of proportions of completed trials during retrieval.

Response	24-hr group	1-wk group
HC Hit	47.3% (26.7%)	33.3% (19.8%)
LC Hit	42.2% (20.2%)	51.5% (20.2%)
Miss	10.0% (10.8%)	15.2% (11.9%)

Supplementary Table 3. Mean and standard deviation of responses at the delayed retrieval test, sorted by recognition accuracy and confidence.

Predictors	*Estimates*	*SE*	*CI*	*df*	*t*	*p*
(Intercept)	60.20	3.08	54.17 – 66.23	56.93	19.56	**<0.001**
Group	-6.87	3.08	-12.90 – -0.84	56.93	-2.23	**0.030**
Time	-4.17	0.83	-5.79 – -2.55	56.51	-5.03	**<0.001**
Group : Time	3.46	0.83	1.84 – 5.09	56.51	4.18	**<0.001**
**Random Effects**						
σ^2	2201.61					
$\tau_{00\ subj}$	529.71					
$\tau_{11\ subj.Time}$	29.36					
$\rho_{01\ subj}$	0.51					
N_{subj}	57					
Observations	13195					
Marginal / Conditional R^2	0.027 / 0.224					

Supplementary Table 4. Fixed and random effects of mixed-effects model with data from Figure 4A. Model: Error ~ group * time + (time | subj)

A. Error ~ schema * group * time + (schema + time | subj)

Predictors	*Estimates*	*SE*	*CI*	*df*	*t*	*p*
(Intercept)	0.01	0.06	-0.10 – 0.12	56.94	0.17	0.862
Schema	0.12	0.02	0.09 – 0.15	55.46	7.63	**<0.001**
Group	-0.13	0.06	-0.24 – -0.02	56.94	-2.24	**0.029**
Time	-0.08	0.02	-0.11 – -0.05	56.67	-5.07	**<0.001**
Schema : Group	0.00	0.02	-0.03 – 0.03	55.46	0.07	0.948
Schema : Time	-0.02	0.01	-0.03 – -0.00	13099.10	-2.15	**0.031**
Group : Time	0.06	0.02	0.03 – 0.09	56.67	4.17	**<0.001**
Schema : Group : Time	0.01	0.01	-0.01 – 0.02	13099.10	1.14	0.254
**Random Effects**						
σ^2	0.75					
$\tau_{00\ subj}$	0.19					
$\tau_{11\ subj.Schema}$	0.01					
$\tau_{11\ subj.Time}$	0.01					
ρ_{01}	-0.02					
	0.51					
N_{subj}	57					
Observations	13195					
Marginal / Conditional R^2	0.042 / 0.249					

B. 1-wk group: Error ~ schema * time + (schema + time | subj)

Predictors	*Estimates*	*SE*	*CI*	*df*	*t*	*p*
(Intercept)	0.14	0.07	0.00 – 0.28	29.00	2.00	0.055
Schema	0.12	0.02	0.07 – 0.16	28.34	5.24	**<0.001**
Time	-0.14	0.03	-0.20 – -0.09	29.12	-5.21	**<0.001**
Schema : time	-0.02	0.01	-0.05 – -0.00	6713.07	-2.25	**0.025**
**Random Effects**						
σ^2	0.84					
$\tau_{00\ subj}$	0.14					
$\tau_{11\ subj.Schema}$	0.01					
$\tau_{11\ subj.Time}$	0.02					
ρ_{01}	-0.27					
	0.76					
N_{subj}	29					
Observations	6764					
Marginal / Conditional R^2	0.034 / 0.193					

C. 24-hr group: Error ~ schema * time + (schema + time | subj)

Predictors	*Estimates*	*SE*	*CI*	*df*	*t*	*p*
(Intercept)	-0.12	0.09	-0.30 – 0.06	27.98	-1.30	0.204
Schema	0.12	0.02	0.08 – 0.16	26.92	5.53	**<0.001**
Time	-0.02	0.01	-0.04 – 0.01	26.51	-1.24	0.225
Schema : Time	-0.01	0.01	-0.03 – 0.01	6392.95	-0.79	0.431
**Random Effects**						
σ^2	0.66					
$\tau_{00\ subj}$	0.24					
$\tau_{11\ subj.Schema}$	0.01					
$\tau_{11\ subj.Time}$	0.00					
ρ_{01}	0.19					
	0.26					
N_{subj}	28					
Observations	6431					
Marginal / Conditional R^2	0.015 / 0.283					

Supplementary Table 5. Fixed and random effects of mixed-effects models with data from Figure 4B. **(A)** Model including both groups. **(B).** Model including 1-wk group. **(C).** Model including 24-hr group.

Predictors	*Estimates*	*SE*	*CI*	*df*	*t*	*p*
(Intercept)	66.68	2.45	61.89 – 71.48	54.73	27.26	**<0.001**
Group	-6.35	2.45	-11.15 – -1.56	54.73	-2.60	**0.012**
**Random Effects**						
σ^2	1882.36					
$\tau_{00\ subj}$	300.63					
N_{subj}	57					
Observations	2680					
Marginal / Conditional R^2	0.018 / 0.153					

Supplementary Table 6. Fixed and random effects of mixed-effects model with data from Figure 5A. Model: gen ~ group + (1 | subj)

Predictors	*Estimates*	*SE*	*CI*	*df*	*t*	*p*
(Intercept)	66.44	2.38	61.77 – 71.11	54.18	27.88	**<0.001**
Group	-7.51	2.38	-12.18 – -2.84	54.18	-3.15	**0.003**
Conf_acc1	-12.44	1.39	-15.17 – -9.71	55.86	-8.93	**<0.001**
Conf_acc2	6.17	0.95	4.30 – 8.04	256.51	6.46	**<0.001**
Group : Conf_acc1	-2.45	1.39	-5.18 – 0.28	55.86	-1.76	0.084
Group : Conf_acc2	-0.53	0.95	-2.40 – 1.34	256.51	-0.56	0.579
**Random Effects**						
σ^2	2254.79					
$\tau_{00\ subj}$	293.41					
$\tau_{11\ subj.Conf\_acc1}$	51.37					
$\tau_{11\ subj.Conf\_acc2}$	3.68					
ρ_{01}	0.86					
	-0.89					
N_{subj}	57					
Observations	6688					
Marginal / Conditional R^2	0.074 / NA					

Supplementary Table 7. Fixed and random effects of mixed-effects model with data from Supplementary Figure 1A. Model: Error ~ group * conf_acc + (conf_acc | subj)

A. Both groups: Error ~ schema * group * conf_bin + (schema + conf_bin | subj)

Predictors	*Estimates*	*SE*	*CI*	*df*	*t*	*p*
(Intercept)	-0.02	0.05	-0.11 – 0.08	56.63	-0.38	0.705
Schema	0.13	0.02	0.09 – 0.17	57.96	6.71	**<0.001**
Group	-0.16	0.05	-0.26 – -0.07	56.63	-3.34	**0.001**
Conf bin	-0.17	0.02	-0.21 – -0.14	51.57	-9.47	**<0.001**
Schema : Group	-0.00	0.02	-0.04 – 0.04	57.96	-0.07	0.941
Schema : Conf bin	-0.04	0.01	-0.06 – -0.01	3755.81	-3.21	**0.001**
Group : Conf bin	-0.03	0.02	-0.06 – 0.01	51.57	-1.61	0.113
Schema : Group : Conf bin	-0.02	0.01	-0.04 – 0.00	3755.81	-1.70	0.088

Random Effects

σ^2	0.75
$\tau_{00\ subj}$	0.13
$\tau_{11\ subj.Schema}$	0.01
$\tau_{11\ subj.Conf\ bin}$	0.01
ρ_{01}	-0.41
	0.86
N_{subj}	57
Observations	6688
Marginal / Conditional R^2	0.086 / 0.230

B. 1-week group: Error ~ schema * conf_bin + (schema + conf_bin | subj)

Predictors	*Estimates*	*SE*	*CI*	*df*	*t*	*p*
(Intercept)	0.15	0.06	0.03 – 0.26	28.71	2.48	**0.019**
Schema	0.13	0.03	0.07 – 0.19	30.23	4.33	**<0.001**
Conf bin	-0.14	0.03	-0.19 – -0.09	26.35	-5.50	**<0.001**
Schema : Conf bin	-0.02	0.02	-0.05 – 0.02	2204.52	-1.07	0.284

Random Effects

σ^2	0.86
$\tau_{00\ subj}$	0.09
$\tau_{11\ subj.Schema}$	0.02
$\tau_{11\ subj.Conf\ bin}$	0.01
ρ_{01}	-0.66
	0.81
N_{subj}	29
Observations	3427
Marginal / Conditional R^2	0.038 / 0.140

C. 24-hour group: Error ~ schema * conf_bin + (schema + conf_bin | subj)

Predictors	*Estimates*	*SE*	*CI*	*df*	*t*	*p*
(Intercept)	-0.18	0.08	-0.34 – -0.03	27.65	-2.28	**0.031**
Schema	0.13	0.02	0.08 – 0.18	27.77	5.46	**<0.001**
Conf bin	-0.19	0.02	-0.24 – -0.15	25.61	-7.85	**<0.001**
Schema : Conf bin	-0.05	0.02	-0.08 – -0.02	1376.13	-3.43	**0.001**
**Random Effects**						
σ^2	0.63					
$\tau_{00\ subj}$	0.17					
$\tau_{11\ subj.Schema}$	0.01					
$\tau_{11\ subj.Conf\ bin}$	0.01					
ρ_{01}	-0.16					
	0.91					
N $_{subj}$	28					
Observations	3261					
Marginal / Conditional R^2	0.064 / 0.278					

Supplementary Table 8. Fixed and random effects of mixed-effects models with data from Supplementary Figure 1B. **(A)** Model including both groups. **(B).** Model including 1-wk group. **(C).** Model including 24-hr group.

Predictors	*Estimates*	*SE*	*CI*	*df*	*t*	*p*
(Intercept)	0.13	0.02	0.10 – 0.17	87.81	7.16	**<0.001**
Error [1st deg.]	-0.20	0.27	-0.73 – 0.32	106.61	-0.76	0.451
Error [2nd deg.]	-0.81	0.20	-1.19 – -0.42	92.31	-4.12	**<0.001**
Time	-0.01	0.02	-0.05 – 0.02	93.50	-0.78	0.437
Group	0.01	0.02	-0.03 – 0.04	87.81	0.29	0.775
Error [1st deg.] : Time	-0.05	0.26	-0.56 – 0.46	107.05	-0.19	0.850
Error [2nd deg.] : Time	0.06	0.18	-0.29 – 0.41	82.24	0.32	0.747
Error [1st deg.] : Group	0.03	0.27	-0.49 – 0.56	106.61	0.13	0.898
Error [2nd deg.] : Group	0.05	0.20	-0.33 – 0.43	92.31	0.25	0.803
Time : Group	0.02	0.02	-0.02 – 0.05	93.50	0.97	0.334
Error [1st deg.] : Time : Group	0.04	0.26	-0.47 – 0.55	107.05	0.14	0.888
Error [2nd deg.] : Time : Group	-0.04	0.18	-0.40 – 0.31	82.24	-0.25	0.806
**Random Effects**						
σ^2			0.02			
$\tau_{00\ subj}$			0.00			
$N_{\ subj}$			57			
Observations			114			
Marginal R^2 / Conditional R^2			0.268 / 0.382			

Supplementary Table 9. Fixed and random effects of a mixed-effects model with a quadratic fit to the data from Supplementary Figure 2. Model: schema ~ poly(Error,2) * Time * Group + (1 | subj)
